# Hierarchical and Dynamic Regulation of Defense-Responsive Specialized Metabolism by WRKY and MYB Transcription Factors

**DOI:** 10.3389/fpls.2019.01775

**Published:** 2020-01-31

**Authors:** Brenden Barco, Nicole K. Clay

**Affiliations:** Department of Molecular, Cellular & Developmental Biology, Yale University, New Haven, CT, United States

**Keywords:** secondary metabolism, plant defense, transcription factor, gene regulatory network, regulon, network motifs, feed-forward loop, feed-back loop

## Abstract

The plant kingdom produces hundreds of thousands of specialized bioactive metabolites, some with pharmaceutical and biotechnological importance. Their biosynthesis and function have been studied for decades, but comparatively less is known about how transcription factors with overlapping functions and contrasting regulatory activities coordinately control the dynamics and output of plant specialized metabolism. Here, we performed temporal studies on pathogen-infected intact host plants with perturbed transcription factors. We identified WRKY33 as the condition-dependent master regulator and MYB51 as the dual functional regulator in a hierarchical gene network likely responsible for the gene expression dynamics and metabolic fluxes in the camalexin and 4-hydroxy-indole-3-carbonylnitrile (4OH-ICN) pathways. This network may have also facilitated the regulatory capture of the newly evolved 4OH-ICN pathway in *Arabidopsis thaliana* by the more-conserved transcription factor MYB51. It has long been held that the plasticity of plant specialized metabolism and the canalization of development should be differently regulated; our findings imply a common hierarchical regulatory architecture orchestrated by transcription factors for specialized metabolism and development, making it an attractive target for metabolic engineering.

## Introduction

Plants are engaged in a continuous co-evolutionary struggle for survival with their pathogens. Although they lack mobile defender cells and an adaptive immune system, they rely on their innate immune system to collectively synthesize hundreds of thousands of ecologically specialized, mostly lineage-specific, preformed and pathogen-inducible metabolites at sites of infection (Dixon and Strack, 2003; Wink, 2003; Weng et al., 2012; Chae et al., 2014). Pathogen-inducible specialized metabolites are synthesized under two primary modes of plant innate immunity—pattern- and effector-triggered immunity (PTI and ETI). PTI depends on signaling networks that identify the non-self microbial invader *via* its conserved microbe-associated molecular pattern molecules (MAMPs), whereas ETI utilizes pathogen-specific virulence effector proteins for pathogen detection (Jones and Dangl, 2006). Specialized metabolism is further dependent on gene regulatory networks (GRNs) that respond to perceived threats by activating defense-responsive transcription factors (TFs) (Clay et al., 2009; Chezem et al., 2017; Barco et al., 2019b) and suppressing TFs involved in growth and development (Lozano-Durán et al., 2013; Fan et al., 2014; Malinovsky et al., 2014; Lewis et al., 2015).

TFs are ultimately responsible for controlling the dynamics and output of gene expression in plant specialized metabolism, and genes encoding specialized metabolic enzymes are often organized into regulons, whereby they come under the control of a limited set of TFs for optimal timing, amplitude, and tissue/pathway-specific expression and subsequent metabolite accumulation (Grotewold, 2005; Hartmann, 2007; Martin et al., 2010; Tohge and Fernie, 2012; Omranian et al., 2015). However, transcription networks that are responsive to external perturbations often contain many TFs with overlapping functions and contrasting regulatory activities, as well as regulons that include diverse targets (e.g., genes encoding other TFs, metabolic enzymes for multiple pathways, and non-enzymatic proteins). GRNs are thus elaborate, supercoordinated forms of organization that connect primary and secondary metabolism, environmental signals, and physiological responses such as growth and defense (Aharoni and Galili, 2011; Baghalian et al., 2014). Subsequently, the ability to engineer novel plant specialized metabolism more often than not produces a frustrating array of unanticipated and undesirable outcomes to the system (Colón et al., 2010; Bonawitz and Chapple, 2013).

Much progress has been made in understanding the finer details of GRN architecture. Central to GRN organization are small sets of recurring regulatory circuits called network motifs (Milo et al., 2002; Shen-Orr et al., 2002). Each motif has been experimentally found to perform specific dynamical functions in gene expression and is wired into the network in such a way that preserves its autonomous functions in natural contexts; thus predictions of network dynamics can be made with simple network motifs of core components without precise knowledge of all of the underlying parameters (Alon, 2007; Gutenkunst et al., 2007). One of the most prevalent network motifs in the GRNs of *Escherichia coli* (Shen-Orr et al., 2002; Ma et al., 2004), *Saccharomyces cerevisiae* (Lee et al., 2002; Mangan et al., 2006), mammalian cells (Odom et al., 2004; Ma'ayan et al., 2005; Boyer et al., 2005), and *Arabidopsis thaliana* (*A. thaliana*) (Jin et al., 2015; Defoort et al., 2018) is the three-component feed-forward loop (FFL), which is composed of two cascaded TFs that interact at a target promoter and jointly determine its rate of transcription (Milo et al., 2002; Mangan and Alon, 2003; Alon, 2007). Depending on the nature of the feed-forward regulation of the target gene (e.g., activation and/or repression, AND- and/or OR-logic gating), the FFL architecture has been shown to exhibit four types of expression dynamics: 1) memory effects of input signals, such as persistence detection (noise filtering), fold-change detection, and dynamics detection (Mangan and Alon, 2003; Goentoro et al., 2009; de Ronde et al., 2012; Lee et al., 2014; Chepyala et al., 2016; Gao et al., 2018); 2) temporal effects of target gene responses such as fast or delayed activation and inhibition, oscillations, and (near-)perfect adaptive pulses (Mangan and Alon, 2003; Mangan et al., 2003; Basu et al., 2004; Basu et al., 2005; Mangan et al., 2006; Cournac and Sepulchre, 2009; Ma et al., 2009; Sontag, 2009; Takeda et al., 2012); 3) amplitude- and pulse-filtering of target gene responses (Shen-Orr et al., 2002; Mangan and Alon, 2003; Mangan et al., 2003; Kaplan et al., 2008); and 4) irreversible switches and transitions (Jaeger et al., 2013; Pullen et al., 2013; Lavenus et al., 2015). FFL circuits can also exhibit two or more dynamical functions in a network, for example, noise-filtering and irreversibility by using OR-logic gating of target gene responses for transcriptional activation at a reduced level and AND-logic gating for maximal transcriptional activation (Pullen et al., 2013).

In animals and E. coli, GRNs for growth and development are defined (Gerstein et al., 2010; modEncode Consortium et al., 2010; Cheng et al., 2011; Niu et al., 2011), plants (Lin et al., 2013; Jaeger et al., 2013; Lavenus et al., 2015; Taylor-Teeples et al., 2015; Joanito et al., 2018; Zhan et al., 2018; Chen et al., 2019), and *E. coli* (Semsey et al., 2007). By contrast, such networks for stress-responsive plant specialized metabolism are still largely defined by individual TFs and their overlapping regulons (Li et al., 2014; James et al., 2017; Yang et al., 2017). Little is known about the hierarchical network motifs that enable multiple TFs with activating and repressive functions to coordinately control the dynamics and output of gene expression and metabolic flux in this context.

**Figure 1:**
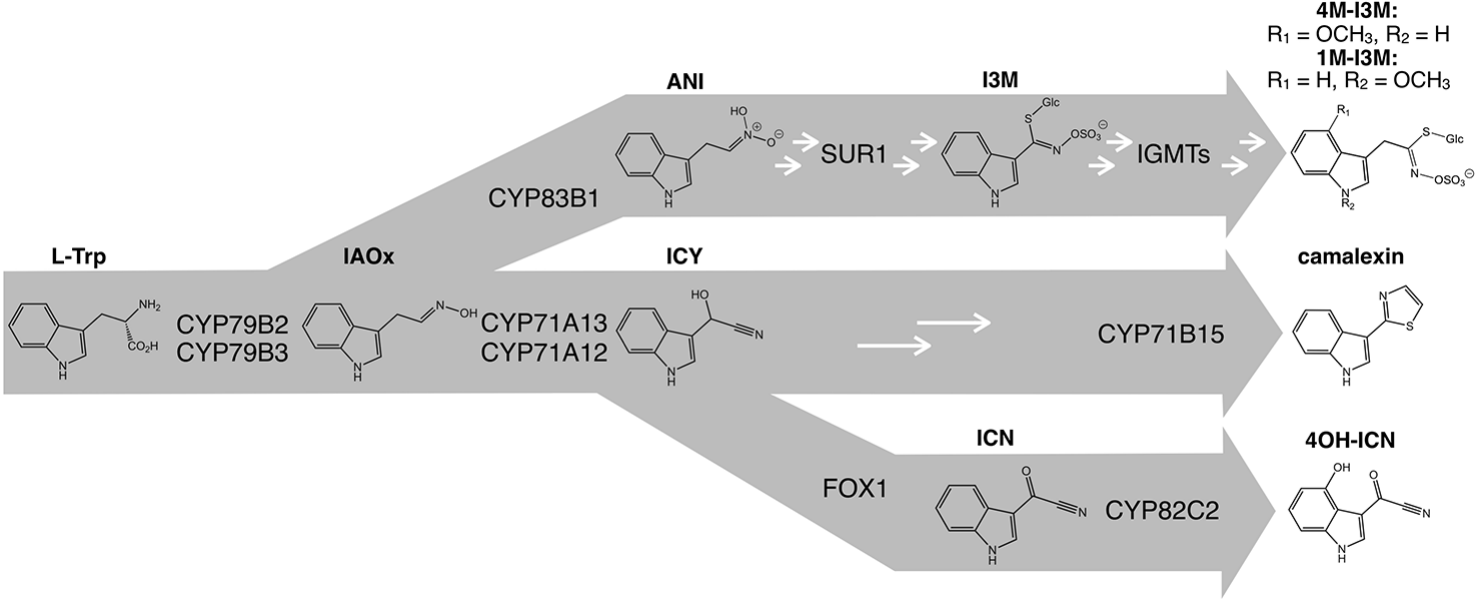
Tryptophan (L-Trp)-derived secondary metabolite pathways in *A. thaliana*. White arrows denote additional enzymatic steps. 1M-I3M, 1-methoxyindol-3-ylmethyl glucosinolate; 4M-I3M, 4-methoxyindol-3-ylmethyl glucosinolate; 4OH-ICN, 4-hydroxyindole-3-carbonylnitrile; ANI, *aci*-nitro indole; I3M, indol-3-ylmethyl glucosinolate; IAOx, indole-3-acetaldoxime; ICN, indole-3-carbonylnitrile; ICY, indole-3-cyanohydrin.

The best-studied defense-responsive specialized metabolites in *A. thaliana* with demonstrated immune functions against fungal and bacterial pathogens are the tryptophan (Trp)-derived camalexin, 4-methoxyindol-3-ylmethyl glucosinolate (4M-I3M), and 4-hydroxyindole-3-carbonylnitrile (4OH-ICN) (Thomma et al., 1999; Ferrari et al., 2003; Bohman et al., 2004; Lipka et al., 2005; Bednarek et al., 2009; Clay et al., 2009; Consonni et al., 2010; Hiruma et al., 2010; Pandey et al., 2010; Sanchez-Vallet et al., 2010; Schlaeppi et al., 2010; Rajniak et al., 2015). 4M-I3M, its immediate precursor 4-hydroxy-I3M (4OH-I3M), and sister metabolite 1-methoxy-I3M are all derived from the parent molecule I3M, and are collectively known as indole glucosinolates (indole GSLs). The biosynthetic pathways of 4M-I3M, camalexin and 4OH-ICN share an early Trp–to–indole-3-acetaldoxime (IAOx) biosynthetic step, courtesy of the genetically redundant cytochrome P450 monooxygenases (CYPs) CYP79B2 and CYP79B3 (Mikkelsen et al., 2000; Glawischnig et al., 2004; Rajniak et al., 2015). CYP71 clade enzymes CYP83B1 and partially redundant CYP71A12/13 respectively convert IAOx to short-lived *aci*-nitro intermediates (ANI) and indole-3-cyanohydrin (ICY) (**Figure 1**) (Bak et al., 2001; Nelson and Werck-Reichhart, 2011; Klein et al., 2013; Rajniak et al., 2015; Barco et al., 2018). CYP71A13 and CYP71B15/PAD3 convert ICY to camalexin, while flavin-dependent oxidase FOX1/AtBBE3 and 4-hydroxylase CYP82C2 convert ICY to 4OH-ICN (**Figure 1**) (Nafisi et al., 2007; Böttcher et al., 2009; Rajniak et al., 2015). 4M-I3M is synthesized from ANI *via* glutathione-*S*-transferases GSTF9–10, γ-glutamyl peptidase GGP1, *S*-alkyl-thiohydroximaste lyase SUR1, UDP-glycosyltransferase UGT74B1, sulfotransferase SOT16, 4-hydroxylases CYP81F1–3, and I3M methyltransferases IGMT1–2 (**Figure 1**) (Chezem and Clay, 2016; Barco et al., 2019a).

The SG12-type R2R3-MYB TFs MYB51 and MYB122 and the Group I WRKY TF WRKY33 are among the best-characterized defense-responsive TFs in *A. thaliana*. MYB51 and MYB122 are activators of 4M-I3M biosynthesis and are required for basal resistance to a variety of bacterial and fungal pathogens (Gigolashvili et al., 2007a; Malitsky et al., 2008; Clay et al., 2009; Humphry et al., 2010; Frerigmann and Gigolashvili, 2014a; Lahrmann et al., 2015; Frerigmann et al., 2016). MYB51 and MYB122 also contribute to camalexin biosynthesis in response to UV stress and the fungal necrotroph *Plectosphaerella cucumerina* through *trans*-activation of *CYP79B2* and *CYP79B3* promoters (Frerigmann et al., 2015; Frerigmann et al., 2016). WRKY33 is an activator of camalexin and 4OH-ICN biosynthesis in response to the ETI-eliciting bacterial pathogen *Pseudomonas syringae* (*Pst*) *avrRpm1* and the fungal necrotroph *Botrytis cinerea* (*B. cinerea*) (Qiu et al., 2008; Birkenbihl et al., 2012; Liu et al., 2015; Birkenbihl et al., 2017; Barco et al., 2019b) and is required for basal resistance to *Pst* and *B. cinerea* (Zheng et al., 2006; Barco et al., 2019b).

To understand how TFs with variable functions and activities coordinately and dynamically govern plant specialized metabolism, we performed temporal studies employing an ETI-eliciting pathogen on host plants exhibiting gain or loss of TF expression. Hydroponically and sterilely grown naïve (unprimed) seedlings were tested to better synchronize the infection process and reduce stress memory effects. We identified a composite hierarchical network motif with WRKY33 as the condition-dependent master regulator and MYB51 as the dual functional regulator that is likely responsible for the gene expression dynamics and metabolic fluxes through the CYP79B2/B3- and CYP82C2-catalyzed steps in the camalexin and/or 4OH-ICN pathways. The characterization of these TF activities in hierarchical gene circuits—in particular how targets are dynamically and coordinately controlled—will better inform how new biosynthetic pathways can be engineered or evolved.

## Results

### MYB51 and MYB122 Are Necessary for 4OH-I3M and 4M-I3M Biosynthesis in ETI

**Figure 2:**
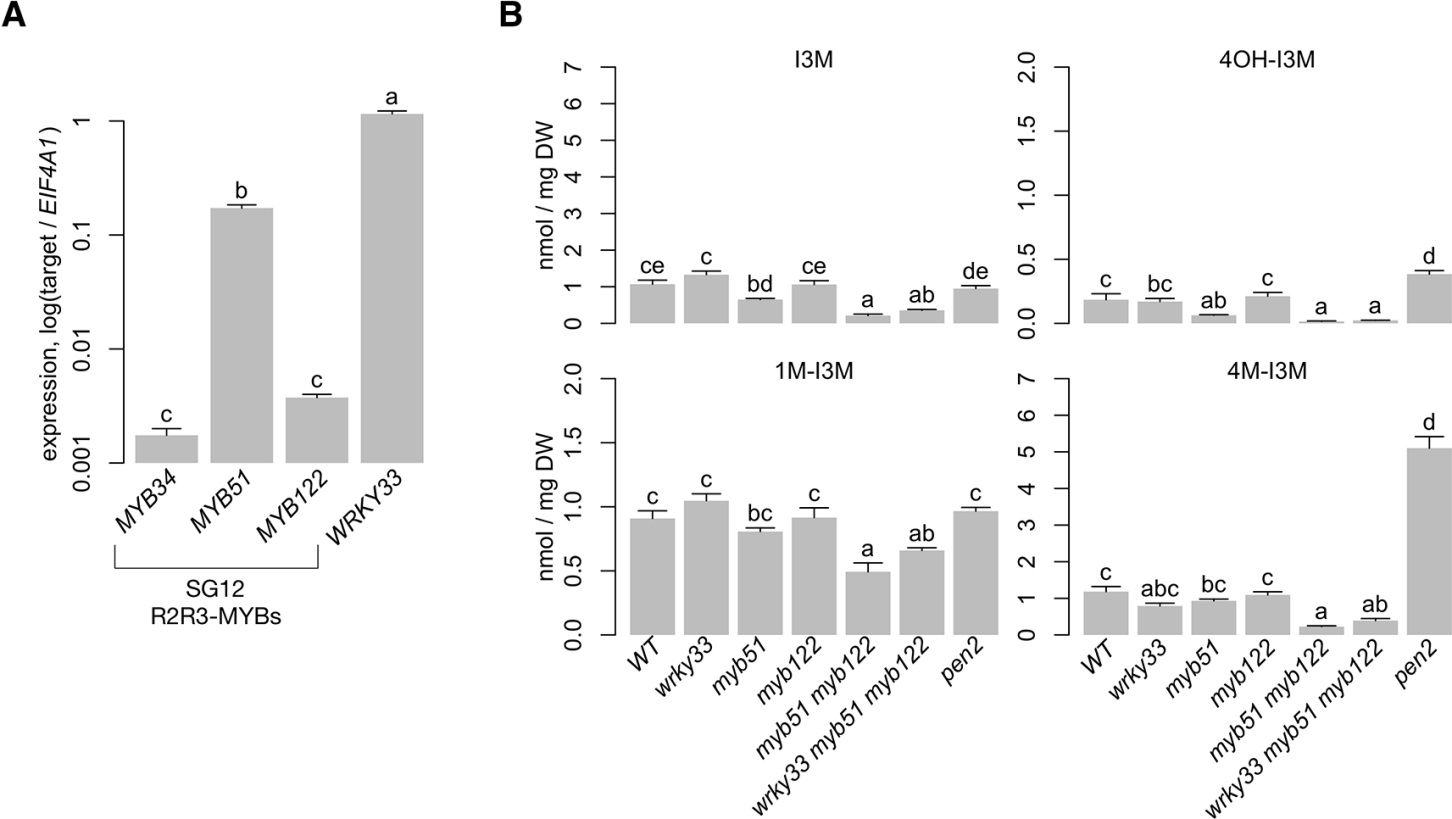
*MYB51 and MYB122* are necessary for 4OH-I3M and 4M-I3M biosynthesis in ETI. **(A)** qPCR analysis of indole glucosinolate (GSL) regulatory genes and WRKY33 in 9-day-old wild-type (WT) seedlings co-elicited with 20 μM dexamethasone (Dex) and *Pseudomonas syringae avrRpm1* (*Psta*) for 12 h. Data represent mean ± SE of four replicates of 13–17 seedlings each. Expression values were normalized to that of the housekeeping gene *EIF4A1*. SG12, subgroup 12 **(B)** HPLC-DAD analysis of I3M, 4-hydroxyindol-3-ylmethyl glucosinolate (4OH-I3M), 4M-I3M, and 1M-I3M in 9-day-old seedlings elicited with *Psta* for 24 h. DW, dry weight. The *pen2* mutant cannot hydrolyze indole GSLs and thus over-accumulates defense-induced 4OH-I3M and 4M-I3M (Bednarek et al., 2009; Clay et al., 2009). Data represent the mean ± SE of four replicates of 13–17 seedlings each. Different letters denote statistically significant differences (*P* < 0.05, one-factor ANOVA coupled to Tukey's test). Experiments were performed twice, producing similar results.

Previous studies have shown that MYB34, MYB51, and MYB122 distinctly regulate indole GSL biosynthesis in response to plant hormones. MYB51 is the central regulator of indole GSL synthesis upon salicylic acid and ethylene (ET) signaling, MYB34 is the key regulator upon abscisic acid (ABA) and jasmonic acid (JA) signaling, and MYB122 has a minor role in JA/ET-induced indole GSL biosynthesis (Frerigmann and Gigolashvili, 2014a). In addition, MYB51 is the major regulator of pathogen-/MAMP-induced 4M-I3M biosynthesis, with MYB122 having a minor role (Clay et al., 2009; Frerigmann et al., 2016). To identify the *A. thaliana* SG12-type R2R3-MYB regulator(s) of 4M-I3M biosynthesis in ETI, we compared the host transcriptional response to PTI-eliciting bacterial MAMP flagellin epitope flg22 with that to ETI-eliciting bacterial pathogen *Pst avrRpm1* (*Psta*) under similar conditions as those of previous studies (Denoux et al., 2008; Clay et al., 2009; Rajniak et al., 2015). *MYB51* and *MYB122* were induced in response to flg22, with *MYB51* expression increasing as high as 50-fold (**Supplementary Table 1**) (Denoux et al., 2008). Similarly, *MYB51* was about 100-fold induced in response to *Psta* (**Figure 2A**). The observed expression of *MYB51* and *MYB122* and quantitative differences in transcriptional responses between flg22 and *Psta* are consistent with previous transcriptional studies of other PTI and ETI elicitors (**Supplementary Table 1**; Tao et al., 2003; Navarro et al., 2004; Toufighi et al., 2005; Austin et al., 2016; Frerigmann et al., 2016).

**Figure 3:**
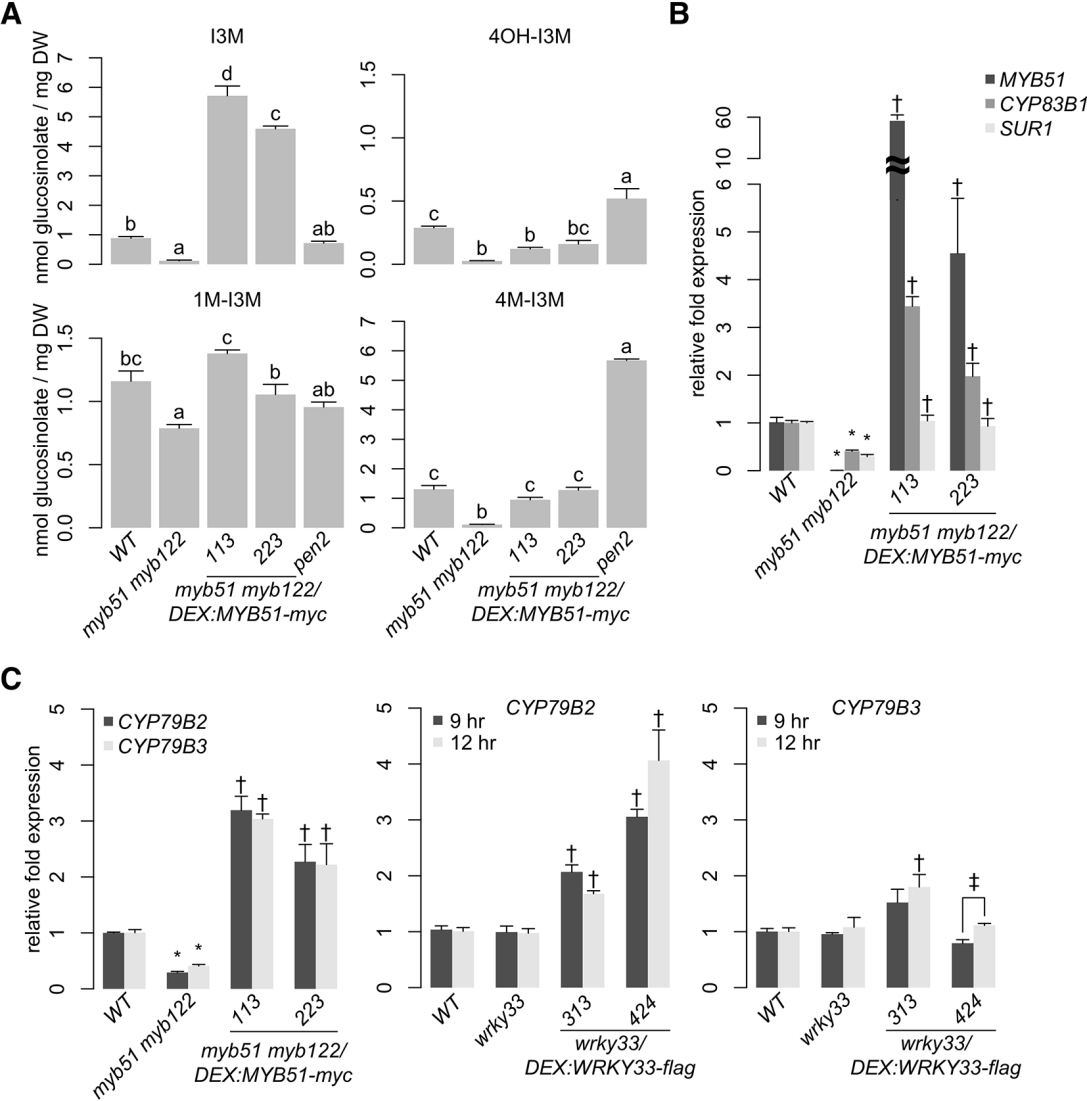
MYB51 and WRKY33 activate IAOx and indole glucosinolate–synthesizing genes. **(A)** HPLC-DAD analysis of I3M, 4OH-I3M, 4M-I3M, and 1M-I3M in 9-day-old seedlings co-elicited with 20 μM Dex and *Psta* for 24 h. The *pen2* mutant cannot hydrolyze indole glucosinolates and thus over-accumulates defense-induced 4OH-I3M and 4M-I3M (Bednarek et al., 2009; Clay et al., 2009). Data represent the mean ± SE of four replicates of 13–17 seedlings each. Different letters denote statistically significant differences (*P* < 0.05, one-factor ANOVA coupled to Tukey's test). Experiments were performed twice, producing similar results. **(B, C)** qPCR analysis of *MYB51*, *CYP83B1*, and *SUR1* **(B)** and of *CYP79B2* and *CYP79B3* **(C)** in 9-day-old seedlings co-elicited with 20 μM Dex and *Psta* for 12 h **(B**, **C**, left**)** or for 9 and 12 h **(C**, middle and right**)**. Data represent mean ± SE of four replicates of 13–17 seedlings each. Expression values were normalized to that of the housekeeping gene *EIF4A1* and are relative to those of 9 h elicited WT plants (**C,** middle and right). Asterisks and daggers denote statistically significant differences compared to wild-type and *myb51 myb122* **(B**, **C**, left**)** or *wrky33* **(C**, middle and right**)**, respectively, and double daggers in **(C)** denote statistically significant differences between 9 and 12 h time points (*P* < 0.05, two-tailed *t* test). DW, dry weight.

To assess functional redundancy between MYB51 and MYB122 in activating 4M-I3M biosynthesis in ETI, we compared the host metabolic response to *Psta* in wild-type (WT), a loss-of-function *myb51* transposon insertion mutant, a newly isolated loss-of-function *myb122-3* T-DNA insertion mutant, and the *myb51 myb122-3* double mutant (**Supplementary Image 1A**). As a technical control, we additionally included the indole GSL hydrolysis-impaired *pen2* mutant, which over-accumulates defense-induced 4OH-I3M and 4M-I3M (Bednarek et al., 2009; Clay et al., 2009). We previously have shown that levels of 4M-I3M and its immediate precursor 4OH-I3M were increased at the expense of the parent metabolite I3M in *Psta*-infected WT plants compared to uninfected WT or *Psta*-infected *rpm1* mutant, which is ETI-deficient when elicited with *Psta* (Bisgrove et al., 1994; Barco et al., 2019b). By contrast, I3M and 4OH-I3M levels were reduced in the *Psta*-infected *myb51* mutant relative to *Psta*-infected WT (**Figure 2B**), consistent with a previous report of reduced flg22-elicited indole GSL biosynthesis in *myb51* (Clay et al., 2009). The *myb122-3* mutation consists of a T-DNA insertion in a region that encodes the DNA-binding R2R3 domain and which is further upstream than that of the loss-of-function *myb122-2* mutant (**Supplementary Image 1A**) (Frerigmann and Gigolashvili, 2014). Additionally, *myb122-3* (hereafter referred to as *myb122*) resembles *myb122-2* in exhibiting WT levels of both *MYB122* transcription upstream of the T-DNA insertion and indole GSL metabolism (**Supplementary Image 1B**; **Figure 2B**) (Frerigmann and Gigolashvili, 2014). In the *Psta*-infected *myb51 myb122-3* mutant (hereafter referred to as *myb51 myb122*), severe reductions in all indole GSLs—including 1-methoxy-I3M (1M-I3M)—were observed (**Figures 2B** and **3A**, **Supplementary Image 2**). Consistent with these results, transcript levels of indole GSL core biosynthetic genes *CYP83B1* and *SUR1* were also reduced in *myb51 myb122* (**Figure 3B**).

To confirm that MYB51 is sufficient to activate 4OH-I3M and 4M-I3M biosynthesis in ETI, we utilized in the *myb51 myb122* background the two-component glucocorticoid-inducible system (Aoyama and Chua, 1997) to generate plants that in the presence of the glucocorticoid hormone dexamethasone (Dex) express a WT copy of the *MYB51* gene with a C-terminal fusion to 6x *c-Myc* (*myb51 myb122/DEX:MYB51-myc*) (**Supplementary Image 3**). Induced expression of *MYB51-myc* increased I3M biosynthesis in the *myb51 myb122* mutant to greater than WT levels by more than fourfold in two independent transgenic lines (**Figure 3A**, **Supplementary Image 2**), enough to fully restore 4M-I3M and 1M-I3M biosynthesis to WT levels (**Figure 3A**, **Supplementary Image 2**). Collectively, these results indicate partial functional redundancy between MYB51 and MYB122, with a predominant role for MYB51 in 4OH-I3M and 4M-I3M biosynthesis in ETI.

### MYB51 Directly Activates Secondary Wall MYB-Responsive Element (SMRE)–Containing *CYP79B2, CYP79B3*, and *CYP83B1* Promoters

The mechanism for the transcriptional regulation of camalexin biosynthesis by MYB51 and MYB122 was previously shown to be centered on *CYP79B2* and *CYP79B3*, (**Figure 1**) (Gigolashvili et al., 2007b; Frerigmann et al., 2015). Previous transient *trans*-activation assay studies with target promoter-*GUS* reporter genes have shown that indole GSL biosynthetic regulators MYB34 and MYB51 (and MYB122 in the case of *CYP83B1*) directly target secondary indole metabolic genes *CYP79B2* and its functionally redundant homolog *CYP79B3* (which are shared by indole GSL/camalexin/ICN pathways) and indole GSL-specific pathway gene *CYP83B1*. By contrast, aliphatic GSL biosynthetic regulators (MYB28, MYB29, and MYB76) directly target aliphatic GSL biosynthetic genes as well as the biosynthetic gene *SUR1* which is shared by both aliphatic and indole GSL pathways (**Figure 1**) (Gigolashvili et al., 2007b; Gigolashvili et al., 2008; Frerigmann et al., 2016).

To confirm that MYB51 *trans*-activates *CYP79B2, CYP79B3*, *CYP83B1*, and *SUR1* expression in ETI, we compared host transcriptional response to *Psta* in WT, *myb51 myb122*, and *myb51 myb122/DEX : MYB51-myc*. Transcript levels of *CYP79B2*, *CYP79B3*, and *CYP83B1* were reduced in *myb51 myb122* relative to WT but restored to greater than WT levels upon induced expression of *MYB51-myc* (**Figures 3B, C**). Interestingly, *SUR1* transcript levels were also reduced in *myb51 myb122* and restored to WT levels upon induced expression of *MYB51-myc* (**Figure 3B**).

**Figure 4:**
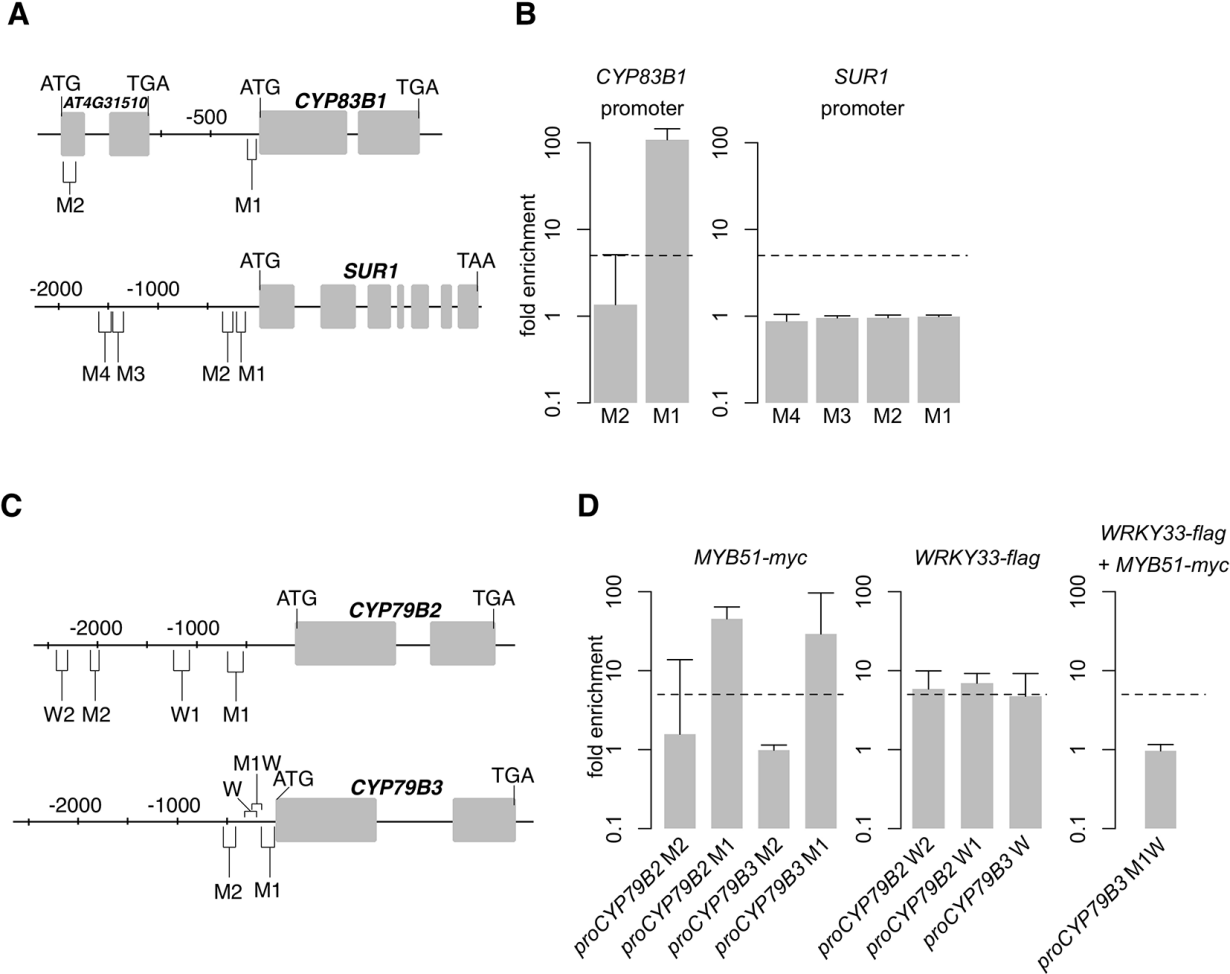
MYB51 and WRKY33 interaction with *CYP83B1*, *CYP79B2*, and *CYP79B3* promoters. **(A–D)** Nucleotide positions **(A, C)**, ChIP-PCR analysis (**B, D**, left and middle), and sequential chromatin immunoprecipitation (ChIP)–PCR analysis (**D**, right) of secondary wall MYB-responsive element (SMRE) (M), W-box (W), and SMRE and W-box (MW)–containing promoter (pro) regions bound by MYB51-myc and/or WRKY33-flag in 9-day-old seedlings co-elicited with 20 μM Dex or mock solution (0.5% DMSO) and *Psta* for 9 h. Boxes in **(A, C)** indicate exons. Dashed lines in **(B**, **D)** represent the fivefold cutoff between weak and strong transcription factor (TF)–DNA interactions. Data in **(B–D)** represent median ± SE of three (**D**, right) and four (**B; D**, left and middle) biological replicates, each containing approximately 200 seedlings. Fold enrichment was determined by calculating the ratio of PCR product intensities in ChIP Dex/Mock to Input Dex/Mock. Experiments were performed twice, producing similar results.

SG12-type R2R3-MYBs are closely related to R2R3-MYBs that bind to type IIG Myb recognition sequences [(T/C)ACC(A/T)A(A/C)C] in electrophoretic mobility shift assays (Romero et al., 1998) and to a shorter 7 bp secondary wall MYB-responsive element (SMRE) consensus sequence [ACC(A/T)A(A/C)(T/C)] within the type IIG Myb recognition sequence in *trans*-activation and chromatin immunoprecipitation (ChIP) assays (Zhou et al., 2009; Zhong and Ye, 2012; Chezem et al., 2017). Moreover, all indole GSL pathway gene promoters contain one or more SMREs (**Figures 4A, C**).

To determine whether MYB51 directly binds to SMRE-containing promoter regions of indole GSL pathway genes, we performed ChIP on 9 h *Psta*-infected mock- and Dex-treated *myb51 myb122/DEX : MYB51-myc* seedlings using antibodies specific to c-Myc (**Supplementary Image 3B**). We then PCR-amplified SMRE-containing regions within 2,000 nt upstream of the translational start site (TSS) of genes encoding the first three enzymes in the indole GSL pathway: *CYP79B2, CYP79B3, CYP83B1*, and *SUR1*. Consistent with a previous report, which utilized transactivation assays (Gigolashvili et al., 2007a), MYB51 bound strongly (> 10-fold enrichment) to the proximal SMRE-containing regions of *CYP79B2* (M1)*, CYP79B3* (M1), and *CYP83B1* (M1) promoters (**Figure 4**; **Supplementary Images 4A, C**; **Supplementary Datasheet 1**). Interestingly, all three proximal SMRE-containing regions contain SMRE motifs that are identical to the three AC elements [AC-I (ACCTACC), AC-II (ACCAACC), and AC-III (ACCTAAC)], which are present in nearly all phenylalanine-derived monolignol pathway gene promoters (Raes et al., 2003). By contrast, MYB51 did not bind to any of the four SMRE-containing regions of the core GSL biosynthetic *SUR1* promoter, three of which contained AC elements (**Figures 4A, B**; **Supplementary Image 4B**; **Supplementary Datasheet 1**), indicating *SUR1* activation by MYB51 is indirect. No indole GSL biosynthetic regulator has yet been shown to directly target core GSL biosynthetic genes (Gigolashvili et al., 2008); our results suggest that MYB51 binds to proximal SMRE-containing promoter regions to directly activate *CYP79B2, CYP79B3*, and *CYP83B1* expression for 4M-I3M biosynthesis in ETI.

### MYB51/MYB122 Regulate Camalexin and ICN Flux-Controlling Genes *CYP79B2/B3*

**Figure 5:**
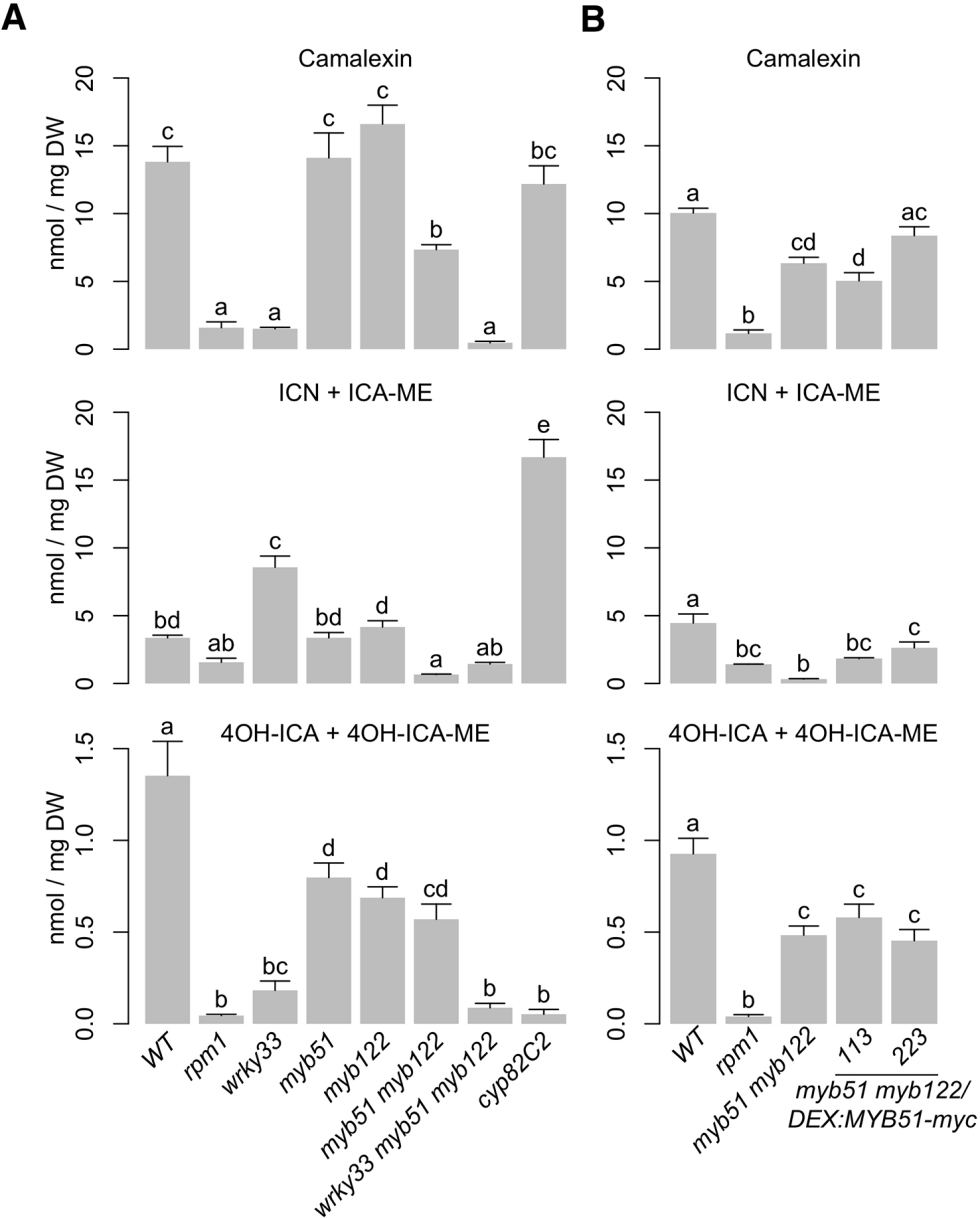
MYB51, MYB122, and WRKY33 regulate metabolic flux through camalexin and 4OH-ICN pathways. **(A, B)** LC-DAD analysis of camalexin (top), ICN (center), and 4OH-ICN (bottom) in 9-day-old seedlings elicited with *Psta* **(A)** or co-elicited with 20 μM Dex and *Psta* **(B)** for 24 h. Data represent mean ± SE of four replicates of 13–17 seedlings each. The *rpm1* mutant is ETI-deficient when elicited with *Psta* (Bisgrove et al., 1994; Barco et al., 2019b). The *cyp82C2* mutant is impaired in 4-hydroxylation of ICN (Rajniak et al., 2015). Different letters denote statistically significant differences (*P* < 0.05, one-factor ANOVA coupled to Tukey's test). Experiments in **(A)** were performed twice, producing similar results. Experiments in **(B)** were performed three times, producing a range of results (**Supplementary Image 6**), the average outcome of which is shown. ICN totals consist of the sum of ICN and methanolic degradation product indole-3-carboxylic acid methyl ester (ICA-ME). 4OH-ICN totals consist of the sum of methanolic and aqueous degradation products 4-hydroxyindole-3-carboxylic acid methyl ester (4OH-ICA-ME) and 4-hydroxyindole-3-carboxylic acid (4OH-ICA), respectively. DW, dry weight.

Metabolic flux though the indole GSL pathway is primarily controlled by enzyme activities at the CYP79B2/CYP79B3-catalyzed step, consistent with theoretical predictions of flux control by the first biosynthetic step (Mikkelsen et al., 2000; Zhao et al., 2002; Sugawara et al., 2009; Wright and Rausher, 2010). In addition, flux though the indole GSL pathway is also likely regulated by SG12-type R2R3-MYBs through changes in *CYP79B2/CYP79B3* gene expression (Celenza et al., 2005; Gigolashvili et al., 2007a; Frerigmann and Gigolashvili, 2014). Recently, MYB51 and MYB122 have been shown to also regulate camalexin biosynthesis in response to flg22 and UV stress (Frerigmann et al., 2015; Frerigmann et al., 2016). Since MYB51 can directly activate *CYP79B2* and *CYP79B3* promoters in response to *Psta* (**Figures 4C, D**, **Supplementary Image 4C**), we hypothesized that MYB51 and MYB122 may also regulate flux through the camalexin and 4OH-ICN pathways through changes in *CYP79B2/CYP79B3* gene expression. To test this hypothesis, we compared the host metabolic and transcriptional response to *Psta* in WT, *myb51, myb122, myb51 myb122*, and *myb51 myb122/DEX : MYB51-myc*. Camalexin levels were largely unchanged in *myb51* and *myb122* single mutants relative to WT, and reduced in the *myb51 myb122* double mutant (**Figure 5**, **Supplementary Image 5**). Similarly, the level of ICN, the immediate precursor to 4OH-ICN, was unchanged in single mutants relative to WT, and nearly abolished in the double mutant, comparable to the ETI-deficient *rpm1* mutant (**Figure 5**, **Supplementary Image 5**). Interestingly, impairments in 4OH-ICN were observed in both *myb51* and *myb122* single and double mutants (**Figure 5**, **Supplementary Image 5**). Induced expression of *MYB51-myc* in at least one line restored ICN biosynthesis on average to WT levels relative to the *myb51 myb122* background (**Figure 5B**, **Supplementary Image 5**). By contrast, camalexin and 4OH-ICN levels were on average not modulated by induction of *MYB51-myc*.

Interestingly, a wide range of responses was observed with respect to ICN and 4OH-ICN induction in *MYB51-myc* lines (**Figure 5B**, **Supplementary Image 6**). Possible explanations for this variability include the chemical instability of ICN and 4OH-ICN as well as imperfect synchronization of the infection process on whole host plants. To better understand the effect of *MYB51-myc* induction on camalexin and 4OH-ICN pathway activity, we investigated the effect of MYB51-myc on expression of genes downstream from *CYP79B2*/*CYP79B3*. In agreement with the reported inability of MYB51 and MYB122 to *trans*-activate the promoters of camalexin biosynthetic genes *CYP71A13* and *CYP71B15* (also known as *PAD3*) (Frerigmann et al., 2015), transcript levels of *CYP71A13* and *CYP71B15* as well as 4OH-ICN biosynthetic genes *CYP71A12* and *FOX1* were repeatedly unchanged or slightly elevated in *myb51 myb122* relative to WT (**Supplementary Image 7**). Furthermore, induced expression of *MYB51-myc* had no effect on *CYP71A12* and *CYP71A13* expression and increased *CYP71B15* and *FOX1* expression in *myb51 myb122* by only 1.5-fold (**Supplementary Image 7**). These results suggest that in ETI, MYB51 and MYB122 regulate biosynthetic flux to camalexin, ICN, and 4OH-ICN through gene expression changes to *CYP79B2/CYP79B3* and not to *CYP71A13, CYP71B15, FOX1,* or *CYP71B15.*

### WRKY33 Regulates Camalexin and ICN Flux-Controlling Genes *CYP79B2/B3*

The TF WRKY33 was recently shown to be a major regulator of camalexin and 4OH-ICN biosynthesis in ETI, directly activating nearly all associated biosynthetic genes in response to *Psta* (**Figure 3C**) (Qiu et al., 2008; Barco et al., 2019b). To determine WRKY33's contribution to flux regulation of camalexin and 4OH-ICN pathways in ETI, we compared host metabolic responses to *Psta* in *wrky33*, *myb51 myb122*, and the newly generated *wrky33 myb51 myb122* triple mutant (**Supplementary Image 1A**). Further reductions in indole GSL levels were not observed in the *wrky33 myb51 myb122* mutant relative to *myb51 myb122* (**Figure 2B**), indicating WRKY33 does not contribute toward flux regulation of 4M-I3M biosynthesis in ETI. In contrast, (4OH-)ICN and camalexin levels, which were reduced to varying degrees in *myb51 myb122* and *wrky33,* were collectively abolished in the *wrky33 myb51 myb122* mutant to levels comparable to the ETI-deficient *rpm1* mutant (**Figure 5A**). These results suggest that WRKY33 also contributes to flux regulation of the camalexin and 4OH-ICN pathways in ETI through changes in CYP79B2/CYP79B3 gene expression.

### WRKY33 Directly Activates W-Box–Containing *CYP79B2 and CYP79B3* Promoters

MYB51 is necessary and sufficient for *Psta*-induced 4OH-I3M and 4M-I3M biosynthesis (**Figures 2B** and **3A**, **Supplementary Image 2**), whereas WRKY33 appears to have no role in their synthesis (**Figure 2B**). On the other hand, WRKY33 is necessary and sufficient for *Psta*-induced camalexin and 4OH-ICN biosynthesis (**Figure 5A**) (Barco et al., 2019b), and MYB51 has a supporting role in their synthesis (**Figure 5**). MYB51 is also necessary and sufficient for *Psta*-induced *CYP79B2* and *CYP79B3* expression (**Figure 3C**). Since all Trp-derived defense metabolites require *CYP79B2* and/or *CYP79B3* for their synthesis, it is likely that WRKY33, like MYB51, also directly activates *CYP79B2* and *CYP79B3* expression in ETI. To test this, we first compared *Psta*-induced *CYP79B2* and *CYP79B3* expression in WT, *wrky33,* and previously characterized *wrky33/DEX : WRKY33-flag* lines (Barco et al., 2019b), which express in the *wrky33* mutant background a Dex-inducible WT copy of *WRKY33* with a C-terminal fusion to FLAG tag. *CYP79B2* and *CYP79B3* expression in *wrky33* was mostly unchanged and occasionally reduced compared to WT in response to *Psta* (**Figure 3C**), but increased two- to fourfold upon induced expression of *WRKY33-flag* (**Figure 3C**). This result is consistent with a previous report of unchanged *CYP79B2* and reduced *CYP79B3* expression in *wrky33* in response to the fungal pathogen *B. cinerea* (Liu et al., 2015) and indicates that although WRKY33 activates *CYP79B2* and *CYP79B3* expression under ETI, MYB51 is the predominant player.

WRKY TFs specifically bind to W-box core sequences [TTGAC(T/C)] (Rushton et al., 2010), and WRKY33 preferentially binds W-boxes that are within 500 nt of the “WRKY33-specific” motif [(T/G)TTGAAT]) (Liu et al., 2015). WRKY33 has been previously shown to bind to the distal W-box–containing promoter region of *CYP79B2* (W2 in **Figure 4**) in response to flg22 (Birkenbihl et al., 2017). To test whether WRKY33 binds *CYP79B2* or *CYP79B3* under ETI, we performed ChIP on 9 h *Psta*-infected mock and Dex-treated *wrky33/DEX : WRKY33-flag* seedlings using antibodies specific to FLAG (Barco et al., 2019b), and PCR-amplified W-box/WRKY33 motif-containing regions within 2,500 nt upstream of *CYP79B2* and CYP79B3's TSSs. WRKY33 bound moderately well (approximately fivefold enrichment) to two W-box/WRKY33 motif-containing promoter regions of *CYP79B2* (W1 and W2) and *CYP79B3* (W) genes (**Figure 4D**; **Supplementary Image 4D**), including the previously reported W2 region (Birkenbihl et al., 2017). Since WRKY33 does not contribute to 4M-I3M biosynthesis (**Figure 2B**), these results suggest that WRKY33 directly activates *CYP79B2* and *CYP79B3* expression to increase metabolic flux to camalexin and 4OH-ICN biosynthetic pathways in ETI.

### MYB51 Directly Represses SMRE-Containing *CYP82C2* Promoter

**Figure 6:**
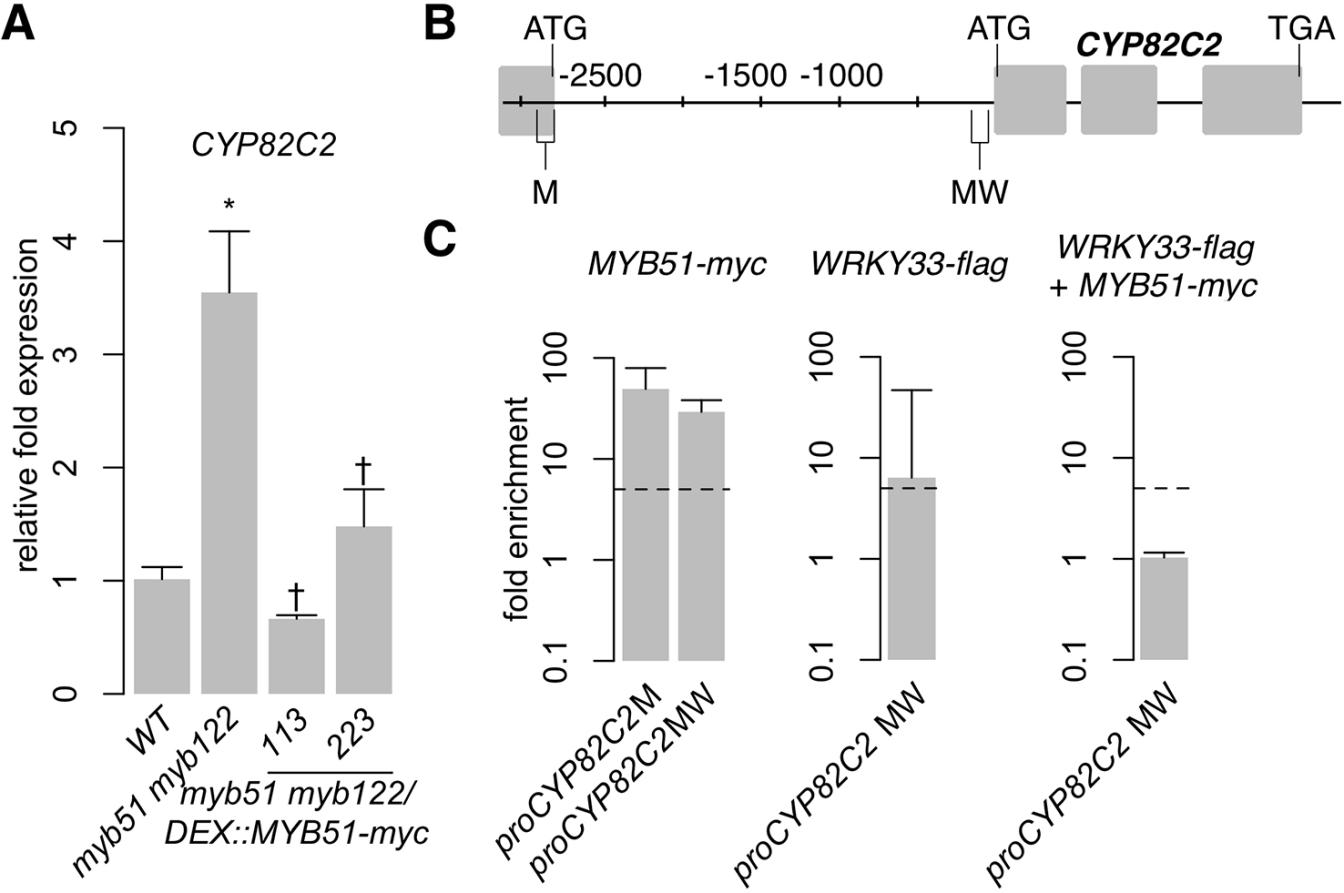
MYB51 directly represses SMRE-containing *CYP82C2* promoter. **(A)** qPCR analysis of *CYP82C2* in 9-day-old seedlings co-elicited with 20 μM Dex and *Psta* for 12 h. Data represent the mean ± SE of four replicates of 13–17 seedlings each. Expression values were normalized to that of the housekeeping gene *EIF4A1* and relative to those of WT plants. Asterisks and daggers denote statistically significant differences compared to wild-type and *myb51 myb122*, respectively (*P* < 0.05, two-tailed *t* test). **(B, C)** Nucleotide positions **(B)**, ChIP-PCR analysis (**C**, left and middle), and sequential ChIP-PCR analysis (**C**, right) of SMRE (M), W-box (W), and SMRE and W-box (MW)–containing *CYP82C2* promoter regions bound by MYB51-myc and/or WRKY33-flag in 9-day-old seedlings co-elicited with 20 μM Dex or mock solution (0.5% DMSO) and *Psta* for 9 h. Dashed lines represent the fivefold cutoff between weak and strong TF–DNA interactions. Data in **(C)** represent the median ± SE of three (right) and four (left and middle) biological replicates, each containing approximately 200 seedlings. Experiments in **(A, C)** were performed twice, producing similar results.

SG12-type R2R3-MYBs have thus far been characterized as transcriptional activators. For example, MYB51 dramatically increases IAOx flux to indole GLSs (**Figure 3A**, **Supplementary Image 2**) by direct activation of *CYP79B2* and *CYP79B3* expression (**Figure 4**; **Supplementary Image 4C**). However, induced expression of *MYB51-myc* leads to variable effects on (4OH-)ICN biosynthesis in *myb51 myb122* (**Figure 5B**, **Supplementary Image 6**). This result led us to hypothesize that additional complex flux regulation of the 4OH-ICN pathway may exist. Consistent with our hypothesis, the *CYP82C2* gene, which encodes an enzyme responsible for the synthesis of 4OH-ICN from ICN (Rajniak et al., 2015), was upregulated 3.5-fold in *myb51 myb122* relative to WT in response to *Psta* (**Figure 6A**). Moreover, induced expression of *MYB51-myc* in *myb51 myb122* decreased *CYP82C2* expression to WT levels (**Figure 6A**), indicating MYB51 represses *CYP82C2* expression. We also observed that MYB51 binds strongly (> 10-fold enrichment) to two SMRE-containing promoter regions of *CYP82C2* (M and MW) (**Figures 6B, C**; **Supplementary Image 8**), indicating *CYP82C2* repression by MYB51 is direct. These results suggest that MYB51 is a dual functional regulator of the 4OH-ICN pathway, directly activating *CYP79B2* and *CYP79B3* expression to increase flux of IAOx to ICN, and directly repressing *CYP82C2* expression to decrease flux of ICN to 4OH-ICN.

### WRKY33 and MYB51 Do Not Co-Localize on *CYP79B3* and *CYP82C2* Promoters

In the course of mapping the *in vivo* binding sites of WRKY33 and MYB51, we observed two overlapping localization patterns. The first one involves the WRKY33-bound W and the MYB51-bound M1 promoter regions of *CYP79B3* (**Figure 4C**). A WRKY33-specific motif (TTTGAAT) in the WRKY33-bound W region is 50 nt from an SMRE-2 motif (ACCAACT) in the MYB51-bound M1 region (**Supplementary Datasheet 1**). The second involves the *CYP82C2* promoter region MW (**Figure 6B**), which is bound strongly by MYB51 in *myb51 myb122/DEX : MYB51-myc* plants and bound moderately (6.4-fold enrichment) by WRKY33 in *wrky33/DEX : WRKY33*-flag plants (**Figure 6C**; **Supplementary Image 8**). The WRKY33 and MYB51-bound MW region contains an SMRE-3 motif (ACCAAAC) that is 113 nt from one of two W-boxes (TTGACC) (**Supplementary Datasheet 1**). These observations suggest that WRKY33 and MYB51 could form a transcriptional complex in response to ETI-eliciting pathogens. To determine whether ETI triggers co-localization of WRKY33 and MYB51 at the same *CYP79B3* and *CYP82C2* promoter regions, we performed sequential ChIP-PCR on 9 h *Psta*-infected, mock and Dex-treated seedlings containing both *DEX : MYB51-myc* and *DEX : WRKY33-flag* transgenes (**Supplementary Image 4E**). We observed enrichment of neither the *CYP79B3* region M1W (encompassing SMRE-1 in M1 and WRKY33 motif in W) (**Figures 4C, D**; **Supplementary Image 4F**) nor the *CYP82C2* region MW amplicons from sequential ChIP of MYB51-myc followed by WRKY33-flag (**Figures 6B, C**; **Supplementary Image 8**). These results indicate that ETI does not trigger stable co-localization of MYB51 and WRKY33 at *CYP79B3* and *CYP82C2* promoter regions, and suggest that WRKY33 and MYB51 likely alternate in binding to the *CYP79B3* M1W and *CYP82C2* MW promoter regions. However, it is also possible that due to lower yields of immunoprecipitated chromatin from the second IP compared to the first IP (Mendoza-Parra et al., 2012), transient or weak interactions such as those derived through competitive binding could be missed with this methodology.

### WRKY33 and MYB51 Form a Hierarchical TF Cascade to Control Trp-Derived Defense Metabolism

**Figure 7:**
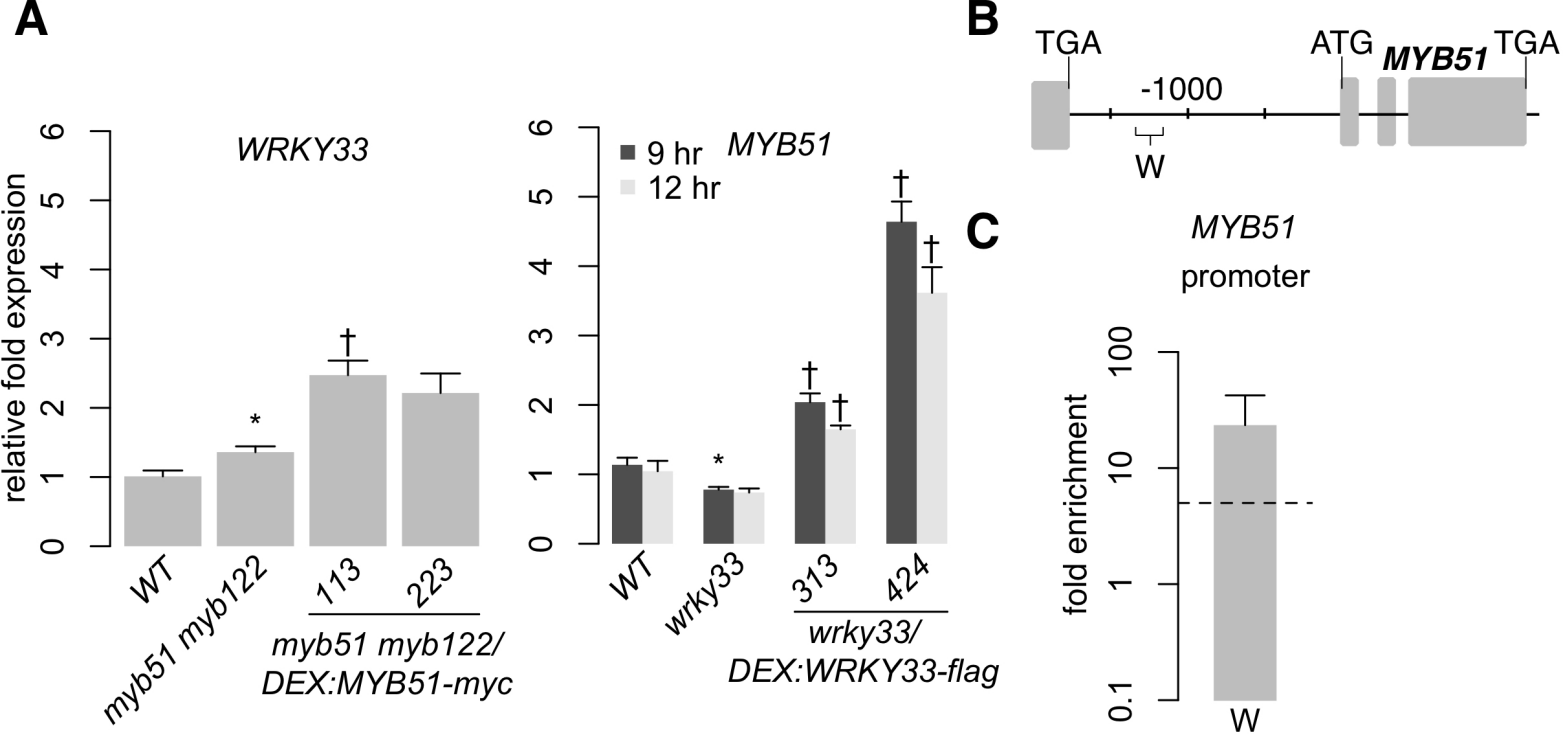
WRKY33 directly activates W-box–containing *MYB51* promoter. **(A)** qPCR analysis of *WRKY33* (left) and *MYB51* (right) in 9-day-old seedlings co-elicited with 20 μM Dex and *Psta* for 12 h (left) or 9 and 12 h (right). Data represent the mean ± SE of four replicates of 13–17 seedlings each. Expression values were normalized to that of the housekeeping gene *EIF4A1* and relative to those of WT plants. Asterisks and daggers denote statistically significant differences compared to wild-type and *myb51 myb122* (left) or *wrky33* (right) respectively (*P* < 0.05, two-tailed *t* test). Experiments were performed at least twice including in WRKY33-myc (Barco et al., 2019b), producing similar results. **(B, C)** Nucleotide positions **(B)** and ChIP-PCR analysis **(C)** of W-box–containing *MYB51* promoter region W bound by WRKY33-flag in *wrky33/DEX:WRKY33-flag* plants co-elicited with 20 μM Dex or mock solution (0.5% DMSO) and *Psta* for 9 h. The *WRKY33* promoter lacks SMRE motifs. Dashed line represents the fivefold cutoff between weak and strong TF–DNA interactions. Data in **(C)** represent median ± SE of four biological replicates, each containing approximately 200 seedlings. Experiments were performed twice, producing similar results.

We observed two overlapping regulatory functions of WRKY33 and MYB51; both TFs activate *CYP79B2/CYP79B3* in response to *Psta* (**Figure 3C**), while WRKY33 activates and MYB51 represses *CYP82C2* expression (**Figure 6A**) (Barco et al., 2019b). The overlapping regulatory functions of WRKY33 and MYB51 suggest that these TFs form a regulatory hierarchy in response to ETI-eliciting pathogens. To determine whether ETI triggers a hierarchical regulatory interaction between WRKY33 and MYB51, we compared *Psta*-induced *WRKY33* and *MYB51* expression in WT, *myb51 myb122*, *myb51 myb122/DEX : MYB51-myc*, *wrky33*, and *wrky33/DEX : WRKY33-flag* plants. *WRKY33* expression was modestly increased in *myb51 myb122* relative to WT, and further increased in at least one *MYB51-myc* line post-elicitation with *Psta* (**Figure 7A**). In addition, these increases in *MYB51-myc* did not rise proportionally with the 4.5- and 50-fold increases in *MYB51-myc* expression (**Figure 3B**), but instead peaked at ~2.5-fold relative to WT level (**Figure 7A**). These results indicate that MYB51 does not directly regulate *WRKY33* expression in ETI. By contrast, *MYB51* expression was reduced in *wrky33* relative to WT, and restored to greater than WT level upon induced expression of *WRKY33-flag* at both 9 and 12 h post-elicitation (**Figure 7A**). Furthermore, the fold increases in *MYB51* expression in *wrky33/DEX : WRKY33-flag* and in similar transgenics expressing a C-terminal c-myc epitope were proportional to the previously reported fold increases in *WRKY33* expression (**Figure 7A**) (Barco et al., 2019b). These results indicate that WRKY33 is necessary and sufficient to activate *MYB51* expression in ETI.

We then performed ChIP-PCR analysis in *wrky33/DEX : WRKY33-flag* lines of the *MYB51* promoter region W, which contains a 50 nt stretch of three W-boxes (**Figure 7B**; **Supplementary Datasheet 1**). We observed strong WRKY33 binding (> 10-fold enrichment) in response to *Psta* (**Figure 7C**; **Supplementary Image 9**), indicating that WRKY33 directly activates the *MYB51* promoter in ETI. This finding is consistent with a previous report of WRKY33 interaction with (but non-regulation of) the *MYB51* locus in response to the fungal pathogen *B. cinerea* (Liu et al., 2015). Since WRKY33 does not contribute to 4M-I3M biosynthesis (**Figure 2B**), these results indicate that a hierarchical TF cascade regulates camalexin and 4OH-ICN biosynthesis in ETI.

### *CYP79B2 *and *CYP79B3* Display Coherent Feed-Forward Loop Connectivity to WRKY33 and MYB51

**Figure 8:**
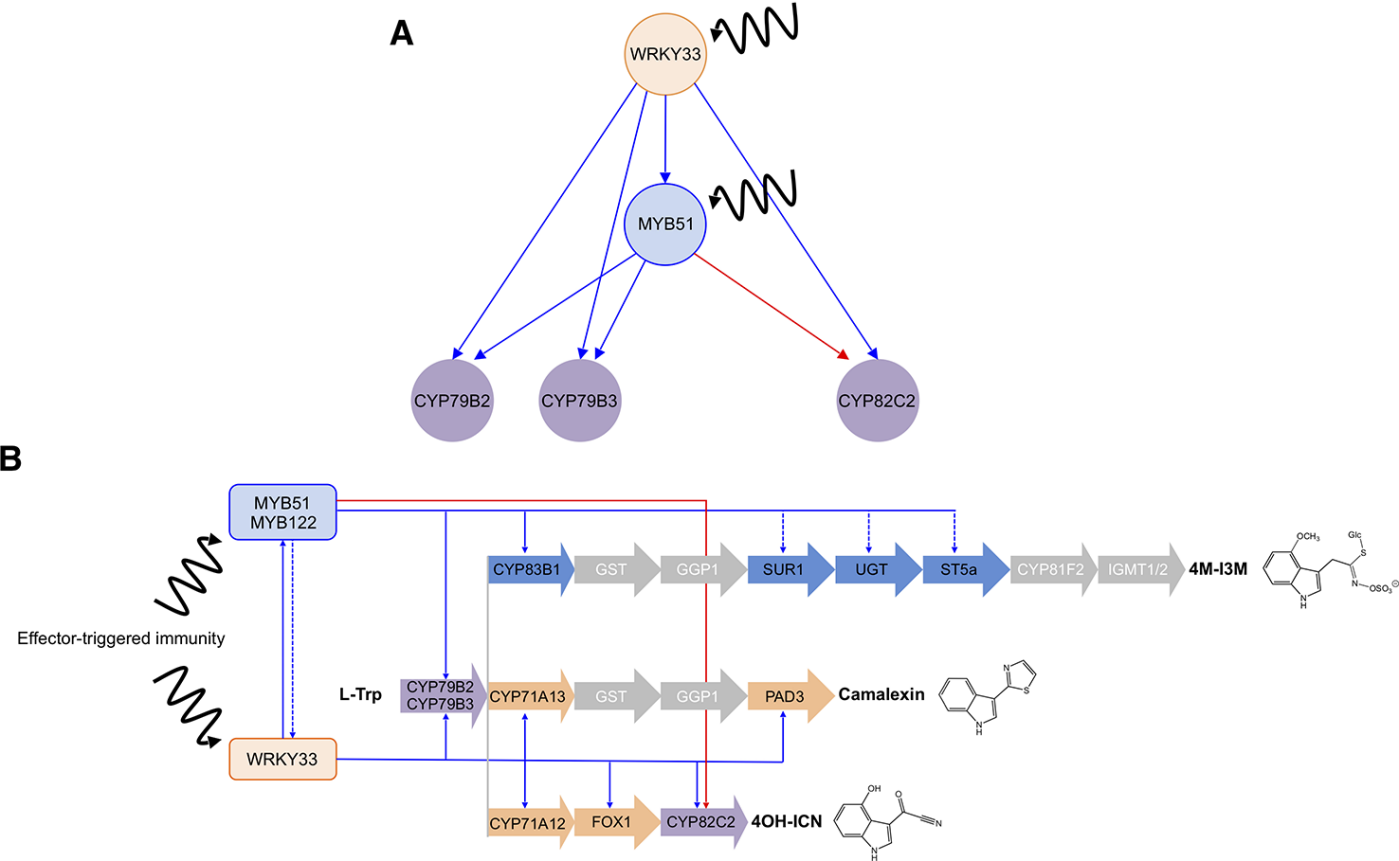
Interlinked coherent and incoherent type 1 feed-forward loops (FFLs) in ETI-responsive specialized metabolism. **(A–B)** Three FFLs identified in this study form a composite hierarchical network motif **(A)** that is likely responsible for the expression dynamics and metabolic flux changes through the camalexin and 4OH-ICN pathways **(B).** Blue and red directed lines indicate activating and inhibitory direct (solid) and indirect (dashed) interactions, respectively, between TFs (orange and blue circles in **A**, rectangles in **B**) and pathway genes (purple circles in **A**, arrows in **B**).

The regulatory interactions between WRKY33, MYB51, and *CYP79B2* (and *CYP79B3*) resemble those of a coherent type 1 FFL circuit (C1-FFL) with OR-gate logic (**Figure 8A**) (Mangan et al., 2003; Alon, 2007), in which WRKY33 activates the target genes *CYP79B2* and *CYP79B3* as well as their activator *MYB51,* and either WRKY33 or MYB51 is sufficient to directly activate *CYP79B2* and *CYP79B3* expression in response to *Psta* (**Figures 3C**, **4** and **7**; **Supplementary Images 4** **and** **9**). The presence of a second transcriptional activator (MYB51) in the indirect regulatory path from the first activator (WRKY33) to the target gene is responsible for a built-in time delay between when the offset signal from the direct path and the offset signal from the indirect path arrive at the target gene, resulting in delayed inactivation (continued activation) of target gene response at the offset of WRKY33 activity (**Figure 8A**) (Mangan et al., 2003; Kalir et al., 2005).

To confirm the hierarchical TF cascade and coherent configuration of the FFL, we compared the temporal dynamics of *MYB51*, *CYP79B2*, and *CYP79B3* expression in two independent lines of *wrky33/DEX : WRKY33-flag* in response to induced expression of *WRKY33-flag* at 9 and 12 h post-elicitation (Barco et al., 2019b). As WRKY33 expression decreased during that time period (Barco et al., 2019b), *MYB51* and *CYP79B2* expression remained steady (**Figures 3C** and **7A**), and *CYP79B3* expression increased in one line (**Figure 3C**). This result indicates continued activation of *CYP79B2* and *CYP79B3* expression in the face of decreasing WRKY33 activity, and confirms their C1-FFL connectivity to WRKY33 and MYB51 in ETI.

### *CYP82C2 *Displays Incoherent Feed-Forward Loop Connectivity to WRKY33 and MYB51

The regulatory interactions between WRKY33, MYB51, and *CYP82C2* resemble those of an incoherent type 1 FFL circuit (I1-FFL) with OR-gate logic (**Figure 8A**) (Milo et al., 2002; Mangan et al., 2003), in which WRKY33 activates both the target *CYP82C2* and its repressor *MYB51,* and either WRKY33 or MYB51 is sufficient to directly regulate *CYP82C2* expression in response to *Psta* (**Figures 6** and **7**; **Supplementary Images 8****–****9**). The I1-FFL motif was shown to have, among other dynamical functions, the ability to produce a non-monotonic (first increasing and then decreasing) target gene response to increasing expression of the activator TF (Basu et al., 2005; Entus et al., 2007; Kaplan et al., 2008). A necessary condition to this non-monotonic behavior (i.e., a concentration-dependent, bell-shaped expression pattern) is that the repressor TF promoter remains responsive to a high level of activator TF activity, regardless of whether the activator and repressor can simultaneously bind the target promoter region (Kaplan et al., 2008).

To confirm the hierarchical TF cascade and incoherent configuration of the FFL, we compared *MYB51* and *CYP82C2* expression between WT and *wrky33/DEX : WRKY33-flag*. *MYB51* expression exceeded WT levels by greater than twofold, and was proportional to fold increases in *WRKY33* expression reported in Barco et al. (2019b) (**Figure 7A**), indicating that the *MYB51* promoter responds monotonically to high (greater than WT) levels of WRKY33 activity. By contrast, *CYP82C2* expression was restored to WT levels by 9 h post-elicitation with *Psta* and either remained steady or decreased to below WT levels by 12 h post-elicitation, and thus was not proportional to fold increases in *WRKY33* expression at both time points reported in Barco et al. (2019b) (**Figure 6A**). These results indicate that the *CYP82C2* promoter responds non-monotonically to high (greater than WT) level of WRKY33 activity, as predicted by its I1-FFL connectivity to WRKY33 and MYB51 in ETI.

### Predominant Role of Camalexin and 4OH-ICN in Bacterial Resistance

**Figure 9:**
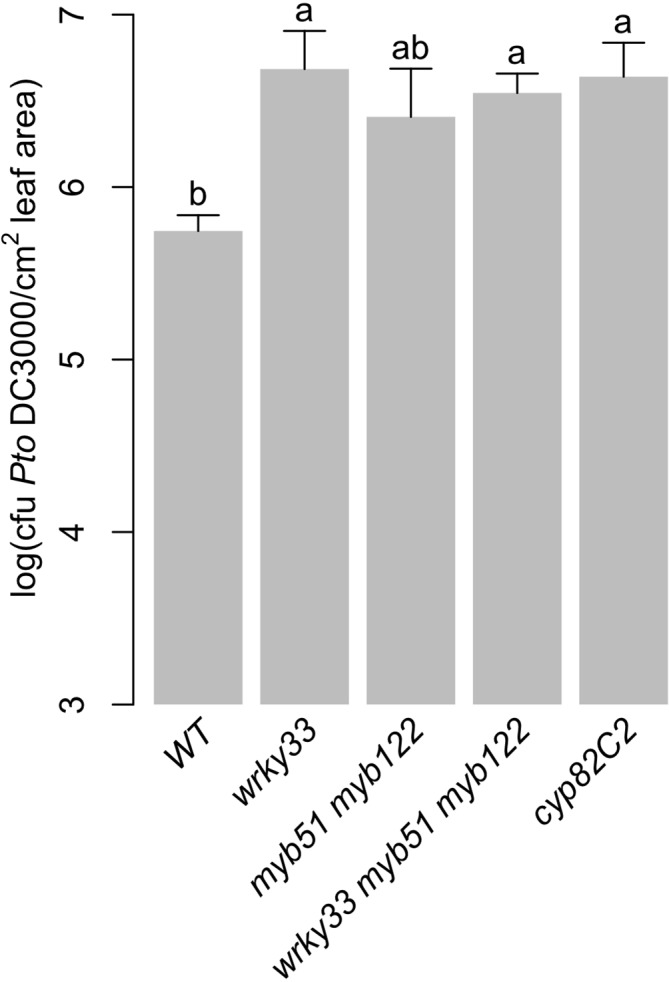
Predominant role of camalexin and 4OH-ICN metabolism in bacterial resistance. Bacterial growth analysis of *Pst* in surface-inoculated 5-week-old leaves. Data represent the mean ± SE of 8–12 biological replicates with one leaf each. Different letters denote statistically significant differences (*P* < 0.05, one-factor ANOVA coupled to Tukey's test). CFU, colony-forming units. Experiments were performed at least twice, producing similar results.

Camalexin and 4OH-ICN have been shown to contribute non-redundantly to basal immunity against *Pst*, with WRKY33 as a major regulator (**Figure 5A**) (Qiu et al., 2008; Rajniak et al., 2015; Barco et al., 2019b). 4M-I3M also contributes to basal immunity against *Pst*, with MYB51/MYB122 as major regulators (**Figures 2B**–**3A**, **Supplementary Image 2**) (Clay et al., 2009). To determine the extent of redundancy between 4M-I3M and camalexin/4OH-ICN in bacterial resistance, we used the TF mutants described in this study as proxies for mutants in defense-induced camalexin/4OH-ICN and 4M-I3M biosynthesis. Specifically, we compared bacterial growth of *Pst* in adult leaves of WT, *wrky33, myb51 myb122*, and *wrky33 myb51 myb122.* Consistent with previous reports, surface-inoculated leaves of *wrky33* and the 4OH-ICN biosynthetic mutant *cyp82C2* mutant showed increased susceptibility to *Pst* relative to WT (**Figure 9**) (Rajniak et al., 2015; Barco et al., 2019b). The *wrky33 myb51 myb122* mutant also showed increased susceptibility to *Pst* relative to WT and comparable to *wrky33* and *cyp82C2* (**Figure 9**). By contrast, the bacterial resistance of *myb51 myb122* was intermediate between WT and *wrky33*, such that it was not significantly different from either line (**Figure 9**). These results suggest a predominant role of camalexin and 4OH-ICN in bacterial resistance.

## Discussion

A signaling node between cell surface receptors and genes underlying specialized metabolism, TFs are also core components of network motifs, whose topological features are often independent from detailed reaction mechanisms and kinetic parameters (Alon, 2007; Gutenkunste et al., 2007) and thus can be easily tuned or adjusted to produce the desired expression dynamics and metabolic output. Despite successes in mapping and modeling GRNs for a wide variety of model systems, only a handful of case studies have linked network architecture in its natural context to its dynamic performance and physiological relevance. Here, we identified two C1-FFLs and one I1-FFL that form a composite hierarchical transcription network motif, with WRKY33 as the condition-dependent master regulator and MYB51 as the dual functional regulator (**Figure 8A**). This composite motif is likely responsible for the observed gene expression dynamics and subsequent metabolic fluxes through the CYP79B2/CYP79B3- and CYP82C2-catalyzed steps in the camalexin and/or 4OH-ICN pathways in ETI (**Figure 8B**). One of the two motifs may have also facilitated the evolution of the 4OH-ICN pathway in *A. thaliana via* regulatory capture of the newly evolved *CYP82C2* gene into the MYB51 regulon (see below).

### Hierarchical Regulatory Architecture of ETI-Responsive Specialized Metabolism

The GRNs of *E. coli*, *C. elegans,* human cells, and *A. thaliana* are characterized by an FFL-enriched hierarchical modularity that is predicted or demonstrated to closely overlap with known environmental stress responses or biological functions (Dobrin et al., 2004; Ma et al., 2004; Tang et al., 2012; Defoort et al., 2018). In addition, most FFLs in the simplest GRN (*E. coli*) contain global regulators, which often act together with local regulators to regulate functionally far-related genes, resulting in a network arrangement of different layers by regulatory depth, with global regulators in the top layers, target genes in the bottom layer, and FFLs spanning the layers (Ma et al., 2004). Consistent with this finding, WRKY33 and MYB51 serve as the respective global and local regulators for the FFL circuits identified in this study, spanning a hierarchical regulatory architecture in the transcriptional network of ETI-responsive specialized metabolism. WRKY33 is characterized as a global regulator because it controls functionally diverse gene modules, such as hormone homeostasis in response to *B. cinerea* (Liu et al., 2015), camalexin and 4OH-ICN biosynthesis in response to flg22, *Psta*, *B. cinerea*, and/or *Pst* (**Figure 5A**) (Qiu et al., 2008; Birkenbihl et al., 2012; Birkenbihl et al., 2017; Barco et al., 2019b), and immune receptor signaling in response to flg22 (Logemann et al., 2013). By contrast, MYB51 is a local regulator, controlling only Trp-derived specialized metabolism in response to flg22, *Psta*, and the fungal pathogen *P. cucumerina* (**Figures 2B**–**3A** and **6**; **Supplementary Image 2**) (Clay et al., 2009; Frerigmann et al., 2016). FFLs in the transcription networks for lignocellulose metabolism in *A. thaliana* and phenolic metabolism in maize have been shown to link developmental regulators to metabolic regulators and their target biosynthetic genes (Zhong and Ye, 2012; Yang et al., 2017), but to our knowledge, the FFLs identified in this study are the first to link two metabolic regulators in plant specialized metabolism.

Compared to the canalization of development, which breaks down under 'pathological conditions' (Waddington, 1942), defense-responsive metabolism has fewer, if any, irreversible regulatory switches, and a greater potential for fine-tuning through rapid interconversion between compounds (Samal and Jain, 2008; Chandrasekaran and Price, 2010; Watson and Walhout, 2014), thus calling into question the necessity of a hierarchical regulatory architecture or fine-tuning through gene expression for defense-responsive metabolism (Li et al., 2014). However, metabolic fluxes through a pathway are often regulated in response to external perturbations (Fell, 1997) through changes in the activities of its enzymes *via* gene expression and/or covalent modification (termed hierarchical regulation), and through changes in their interactions with other enzymes *via* allosteric regulation and/or (co-)substrate saturation (termed metabolic regulation) (ter Kuile and Westerhoff, 2001; Rossell et al., 2005). Hierarchical regulation through proportionate increases in relevant gene expression has been demonstrated for enzymes of central carbon metabolism in response to environmental stresses in single-celled organisms (ter Kuile and Westerhoff, 2001; Even et al., 2003; Daran-Lapujade et al., 2004; Rossell et al., 2005; Rossell et al., 2006). However, to our knowledge, similar studies in defense-responsive plant specialized metabolism have been limited to identifying enzymes with high flux control (Olson-Manning et al., 2013). Our findings suggest significant hierarchical regulation of fluxes through the camalexin and 4OH-ICN pathways through coherent feed-forward activation of the *CYP79B2/CYP79B3* promoters (**Figures 3C**, **4** and **7**; **Supplementary Images 4** **and** **9**; Barco et al., 2019b), and incoherent feed-forward regulation of the *CYP82C2* promoter in response to *Psta* (**Figures 6** and **7**; **Supplementary Images 8****–****9**). Future research is needed to determine whether the CYP82C2 enzyme has high flux control of the 4OH-ICN pathway and to what extent metabolic fluxes through individual enzymes with high flux control are regulated by gene expression or by metabolic regulation in ETI-responsive specialized metabolism. Increased understandings of flux control and regulation would promote systems biological approaches toward engineering plant specialized metabolism (Baghalian et al., 2014).

### Physiological Relevance of FFLs

The three branches of ETI-responsive Trp-derived specialized metabolism are distinctly and tightly controlled, both in timing and amplitude, despite sharing the CYP79B2 and CYP79B3-catalyzed first step (Mikkelsen et al., 2000; Glawischnig et al., 2004; Rajniak et al., 2015). For instance, pathogen defense-responsive indole GSLs have been shown previously to be present at low levels in uninfected plants and accumulate to modest levels at the expense of the parent metabolite I3M in flg22- and *Psta*-inoculated plants (Clay et al., 2009; Barco et al., 2019b), whereas camalexin and 4OH-ICN are absent in uninfected plants, at low-to-undetectable levels in flg22-inoculated plants, and at high levels in *Psta*-inoculated plants (**Figures 2B**–**3A** and **5**; **Supplementary Image 2**) (Rajniak et al., 2015; Barco et al., 2019b). The dynamical functions of three FFLs (two C1-FFLs and one I1-FFL) identified in this study likely contribute to these observed physiological responses.

First, C1-FFLs are proven mechanisms for delaying response times in transcription networks (Mangan and Alon, 2003; Mangan et al, 2003b; Kalir et al., 2005). The C1-FFL connectivity of *CYP79B2* and *CYP79B3* to WRKY33 and MYB51 (**Figure 8A**) is likely responsible for the continued activation of *CYP79B2* and *CYP79B3* expression despite decreasing WRKY33 activity in ETI, which in turn may account for the high production of all defense-responsive Trp-derived metabolites at the onset of ETI, and the continued production of indole GSLs at the offset of ETI. Future experiments are needed to confirm that the duration of the time delay displayed by the two clustered C1-FFLs can be fine-tuned through changes in MYB51's biochemical parameters (for example, its activation threshold for the target gene promoters) (Alon, 2007).

Second, I1-FFLs are proven mechanisms for non-monotonic time-responses in transcription networks that mediate trade-offs in metabolism (Weickert and Adhya, 1993; Kaplan et al., 2008; Ascensao et al., 2016). For example, an I1-FFL was shown to regulate the non-monotonic target gene responses of galactose metabolism so that when cells are severely starved for glucose, galactose breakdown is reduced, and galactose is redirected toward cell wall synthesis (Weickert and Adhya, 1993). Similarly, the I1-FFL connectivity of *CYP82C2* to WRKY33 and MYB51 (**Figure 8A**) is likely responsible for the initial increased and then decreased activation of *CYP82C2* expression in the face of increasing WRKY33 activity in ETI, so that when WRKY33 activity is high, 4OH-ICN synthesis is reduced, and IAOx is redirected toward indole GSL biosynthesis (**Figure 8B**). Indole GSL biosynthesis has been shown to be metabolically linked to auxin homeostasis; severe reductions in indole GSL biosynthesis by mutagenesis has been shown to result in overflow of IAOx to indole-3-acetic acid (auxin) and ultimately to high-auxin phenotypes, such as severe defects in plant growth and development (Boerjan et al., 1995; Bak and Feyereisen, 2001; Mikkelsen et al., 2004). Future experiments are needed to confirm that the concentration-dependent, bell-shaped target gene expression pattern can be fine-tuned through changes in MYB51 concentration and/or MYB51's binding affinity for the target gene promoter (Entus et al., 2007).

### WRKY33 Is a Condition-Dependent Master Regulator

A hierarchical regulatory architecture of ETI-responsive specialized metabolism requires that a single master or key regulator is able to be both necessary and sufficient to drive biosynthesis (Li et al., 2014). Our data show that WRKY33 fulfills that requirement; its absence results in the near-absence of camalexin and 4OH-ICN biosynthesis in *Psta*-infected plants, comparable to the ETI-deficient *rpm1* mutant (**Figure 5A**), and its induced expression restores camalexin and 4OH-ICN biosynthesis in the *Psta*-infected *wrky33* mutant to levels that exceed WT (Barco et al., 2019b). Furthermore, WRKY33 initiates a feed-forward regulation of camalexin and 4OH-ICN biosynthetic gene responses *via* MYB51 (**Figures 3C**, **4**, **6**, and **7**; **Supplementary Images 4****,** **8****, and** **9**). However, WRKY33's ability to initiate camalexin and 4OH-ICN biosynthetic gene responses occurs only in cells undergoing ETI-specific reprogramming (**Figure 5A**) (Barco et al., 2019b). This condition-dependent regulation is consistent with recent ChIP-Seq and expression analysis revealing that WRKY33 binds to a large number of genomic loci in *B. cinerea* or flg22-elicited plants, yet only activates or represses transcription at a subset of these genes (Liu et al., 2015; Birkenbihl et al., 2017). Condition-dependent regulation has also been demonstrated for the classic master regulator in development, MyoD, which initiates skeletal muscle-specific gene expression only in cells conditioned to be permissive to myogenesis (Aziz et al., 2010). Further protein interaction studies are needed to determine mechanistically what additional interactions are needed to activate WRKY33-bound promoters. The simplest hypothesis would be that two distinct classes of chromatin proteins are recruited to WRKY33-bound promoters, and that chromatin remodeling is needed for dynamic switching of gene expression during ETI.

### MYB51 Is a Dual Functional Regulator

Few plant TFs have been identified to act as transcriptional activators and repressors, depending on DNA-binding sequences or interactions with additional co-factors. They include WUSCHEL in stem cell regulation and floral patterning, WRKY53 in leaf senescence, WRKY6 and tomato Pti4 in pathogen defense, and WRKY33 in camalexin and ABA biosynthesis (Robatzek and Somssich, 2002; Miao et al., 2004; González-Lamothe et al., 2008; Ikeda et al., 2009; Liu et al., 2015). Our data show that MYB51 also possesses dual functionality, acting as an activator and repressor in a manner dependent on promoter context. 4OH-ICN and camalexin profiles (**Figure 5**, **Supplementary Images 5****–****6**) and *CYP79B2, CYP79B3*, and *CYP82C2* transcript profiles (**Figures 3C**, **6A**) in *MYB51* gain- or loss-of-function lines—especially the variability in metabolism observed in *myb51 myb122/DEX:MYB51-myc*—indicate complex regulatory control of 4OH-ICN and camalexin biosynthesis by MYB51, in case of 4OH-ICN at both the first and last steps of biosynthesis (**Figure 8B**). By contrast, 4M-I3M metabolite and *CYP83B1* and *SUR1* transcript profiles in *myb51 myb122* and *myb51 myb122/DEX : MYB51-myc* indicate straightforward positive regulation of indole GLS biosynthesis by MYB51 (**Figure 8B**). Further protein interaction studies are needed to determine mechanistically how MYB51 exerts its dual regulatory functions. The simplest hypothesis would be that MYB51 is recruited to distinct repressor and activator complexes at defined promoter sites.

### Regulatory Capture of Newly Duplicated Gene *CYP82C2* Into the MYB51 Regulon

Recent phylogenetic decomposition analysis of the *A. thaliana* GRN indicated that novel genes are more likely to be regulated by conserved TFs in FFLs, as well as attach to gene modules with specific biological functions, instead of forming modules on their own (Defoort et al., 2018). A few newly evolved plant specialized metabolic pathways have been shown to be regulated by existing TFs. They include the *A. thaliana–*specific benzoyloxy-GSL pathway by the Brassicaceae-specific SG25-type R2R3-MYBs MYB115 and MYB118 (Zhang et al., 2015; Barco et al., 2019a), the Brassicales-specific core GSL pathway by the plant lineage–specific SG3e-type MYCs MYC2–5 (Schweizer et al., 2013; Frerigmann et al., 2014b; Chezem and Clay, 2016); and the Brassicaceae-specific camalexin and *A. thaliana*–specific 4OH-ICN pathways by the plant lineage–specific WRKY33 (Bednarek et al., 2011; Rinerson et al., 2015; Schluttenhofer and Yuan, 2015; Barco et al., 2019b). *MYB51/MYB122* and *CYP79B2/CYP79B3* are unique to the GSL-synthesizing plant order Brassicales (Fahey et al., 2001; Bekaert et al., 2012; Barco et al., 2019a), whereas *CYP82C2* is a newly duplicated enzyme gene in the *A. thaliana*–specific 4OH-ICN biosynthetic pathway (Rajniak et al., 2015; Barco et al., 2019b). Our data show that the indole GSL regulator MYB51 interacts with the *CYP82C2* promoter at the M and MW regions (**Figures 6B, C**; **Supplementary Image 8**) and negatively regulates its expression (**Figure 6A**). Further phylogenetic and syntenic analyses are needed to determine mechanistically how the *CYP82C2* gene was recruited into the MYB51 regulon. The simplest hypothesis would be that the *CYP82C2* promoter acquired one or more MYB51-binding sites through mutation and/or transposition (Wittkopp and Kalay, 2012).

## Methods

### Plant Materials and Growth Conditions

For transcriptional and metabolomics analyses, seeds of *A. thaliana* accession Columbia-0 (Col-0) were surface-sterilized in 20% (v/v) bleach/0.1% (v/v) Tween-20 aqueous solution for 5 min, washed three times with sterile water, stratified at 4°C for 2 days, and sown in 12-well microtiter plates sealed with Micropore tape (3M, St. Paul, MN), each well containing 15 ± 2 seeds and 1 ml of filter-sterilized 1X Murashige and Skoog (MS; Murashige & Skoog, 1962) media (pH 5.7–5.8) [4.43 g/L MS basal medium with vitamins (Phytotechnology Laboratories, Shawnee Missions, KS), 0.05% MES hydrate, 0.5% sucrose). The plates were placed on grid-like shelves over water trays on a Floralight cart, and plants were grown under long-day conditions [16 h light cycle (70–80 μE m^−2^ s^−1^ light intensity), 21°C, 60% relative humidity]. For ChIP analyses, approximately 200 surface-sterilized seeds were sown in a 100 × 15 mm petri plate containing 20 ml of 1X MS media. Media were refreshed on day 9 prior to bacterial elicitation. Nine-day-old seedlings were inoculated with *Psta* to OD_600_ of 0.013, and seedlings and/or ~1 ml media were snap-frozen in liquid nitrogen 9 h post-infection for ChIP analyses, 12 h post-infection for qPCR analyses, and 24 to 48 h post-infection for LC-DAD-FLD-MS analyses, prior to −80°C storage.

For bacterial infection assays, plants were grown on soil [3:1 mix of Farfard Growing Mix 2 (Sun Gro Horticulture, Vancouver, Canada) to vermiculite (Scotts, Marysville, OH)] at 22˚C daytime/18˚C nighttime with 60% humidity under a 12 h light cycle [50 (dawn/dusk) and 100 (midday) μE m^−2^ s^−1^ light intensity].

The following homozygous Col-0 mutants and T-DNA/transposon insertion lines were obtained from the Arabidopsis Biological Resource Center (ABRC): *cyp82C2* (GABI _261D12; CS425008; Rajniak et al., 2015); *myb51* (SM_3_16332; CS104159; Clay et al., 2009), *myb122* (also referred to as *myb122-3*, SALK_022993); *pen2* (GABI_134C04; Lipka et al., 2005), *rpm1* (CS67956; Bisgrove et al., 1994), and *wrky33* (SALK_006603, Zheng et al., 2006).

### Plant Binary Vector Construction and Transformation

To generate the *DEX : MYB51-myc* construct, the *MYB51* coding sequence was PCR-amplified from genomic DNA using the primers MYB51gXhoF (5'-AACTCGAGATGGTGCGGACACCGTG-3') and MYB51gStuR (5'-AAGGCCTCCAAAATAGTTATCAATTTCGTC-3'), and subcloned into the *Xho*I/*Stu*I sites of pTA7002-6x c-Myc binary vector (Aoyama and Chua, 1997; Chezem et al., 2017). The construct was introduced into *myb51 myb122-3* plants *via Agrobacterium tumefaciens*–mediated floral dip method (Clough & Bent, 1998), and transformants were selected on agar media containing 15 μg/ml hygromycin B (Invitrogen, Carlsbad, CA).

### Extraction and LC-DAD-FLD-MS Analysis of Glucosinolates

Glucosinolates were analyzed as desulfoglucosinolates as previously described by Kliebenstein et al. (2001) with some modifications. Briefly, a 96-well 0.45 μm PVDF filter plate (EMD Millipore, Billerica, MA) was charged with 45 mg DEAE Sephadex A25 (GE Heathcare) and 300 μl of water per well and equilibrated at room temp for 2 h. Prior to sample homogenization, the plate was centrifuged at 400×g for 1 min to remove the water. The homogenate was extracted with 500 μl 70% (v/v) aqueous methanol at 67.5˚C for 10 min and centrifuged at 16,000×g for 2 min. Added to the supernatant was 3 μl of internal standard [IS; 1.25 mM sinigrin (Sigma-Aldrich) in 80% (v/v) ethanol] per mg sample dry weight. Extract was applied to and incubated on the ion exchanger for 10 min. The sephadex resin was washed three times with 70% (v/v) methanol, three times with distilled deionized water (ddH_2_O), and two times with 20 mM sodium acetate (pH 5). Twenty microliters of 25 mg/ml aryl sulfatase (Type H1 from *Helix pomatia*, Sigma-Aldrich) was applied to and incubated on the sephadex resin at RT overnight (Hogge et al., 1988). The plate was centrifuged at 400×g for 1 min, and desulfoglucosinolates were eluted from the sephadex resin by two 100 μl washes with 60% (v/v) methanol and two 100 μl washes with ddH_2_O. Eluate volume was reduced to 250–350 μl using an evaporator. Samples were separated using the gradient shown in **Supplementary Table 2**. A coupled DAD-3000RS diode array detector, FLD-311 fluorescence detector (Dionex), and MSQPlus mass spectrometer collected UV absorption spectra at 229 nm, fluorescence spectra at 275/350 nm (ex/em), and ESI mass spectra in positive/negative ion modes at 100–1,000 m/z, respectively. Glucosinolates were quantified using integrated areas of desulfoglucosinolates in the UV chromatographs at 229 nm and published response factors (Clarke, 2010) for I3M [retention time (RT) = 14.7 min], 1M-I3M (RT = 20.0 min), 4OH-I3M (RT = 9.6 min), and 4M-I3M (RT = 17.3 min).

### Extraction and LC-DAD-FLD-MS Analysis of Camalexin and 4OH-ICN

Ten-day-old seedlings were snap-frozen, lyophilized, weighed, and homogenized using a 5 mm stainless steel bead and ball mill (20 Hz, 4 min). For phytoalexin analysis, homogenate was extracted with 300 μl 80% (v/v) aqueous methanol containing 0.08% (v/v) formate and 2.5 μl IS [200 μM 4-methoxyindole/4M-I (Sigma-Aldrich) in 100% methanol] per mg sample dry weight. For media samples, 2.5 μl of IS was added to extract per mg dry weight of accompanying seedling tissue sample and no formic acid was added to the mobile phases during extraction. Extracts were sonicated for 5 min and centrifuged at 16,000×g for 2 min. The supernatant was filtered using a 0.45 μm polypropylene filter plate (GE Healthcare, Chicago, IL). Samples were separated by reversed-phase chromatography on an Ultimate 3000 HPLC (Dionex, Sunnyvale, CA) system, using a 3.5 μm, 3×150 mm Zorbax SB-Aq column (Agilent, Santa Clara, CA); volume injected was 10 μl. The gradient is shown in **Supplementary Table 2**. A coupled DAD-3000RS diode array detector (Dionex) collected UV absorption spectra in the range of 190–560 nm, a FLD-311 fluorescence detector (Dionex) collected fluorescence data at 275 nm excitation and 350 nm emission, and an MSQPlus mass spectrometer (Dionex) collected ESI mass spectra in positive and negative ion modes in the range of 100–1,000 m/z. Total ICN, 4OH-ICN, and camalexin amounts were quantified using standard curves of standards prepared in *cyp79B2 cyp79B3* seedling extract and integrated areas in the UV chromatographs at 260 nm for 4M-I (RT = 7.7 min); 340 nm for ICN (RT = 11.5 min); 280 nm for ICN degradation product ICA-ME (RT = 9.5 min); and co-eluting 4OH-ICN degradation products 4OH-ICA and 4OH-ICA-ME (RT = 10.1 min); and 320 nm for camalexin (RT = 12.1 min).

### RNA Extraction and qPCR Analysis

Total RNA extraction and qPCR were performed as described in Chezem et al. (2017). The Pfaffl method (Pfaffl, 2001) and calculated primer efficiencies were used to determine the relative fold increase of the target gene transcript over *EIF4A1* (*AT3G13920*) housekeeping gene transcript for each biological replicate. Primer sequences and efficiencies are listed in **Supplementary Table 3**.

### Total Protein Extraction, SDS-PAGE, and Western Blotting

Total protein extraction was performed as previously described (Barco et al., 2019b). Extract [5 µl (DEX : MYB51-myc or DEX : WRKY33-flag) or 50 µl (DEX : MYB51-myc and DEX : WRKY33-flag)] was loaded onto a 10% SDS-PAGE gel, and the separated proteins were transferred to PVDF membrane (Millipore, Billerica, MA), stained with Ponceau S for labeling of total protein, and probed with either FLAG M2 (Sigma-Aldrich, cat# F1804) or c-Myc 9E10 (Santa Cruz Biotechnology, cat# sc-40) antibodies diluted 1:1,000 in 1X PBS containing 5% (w/v) nonfat milk.

### Chromatin Immunoprecipitation and PCR

ChIP was performed on *myb51/DEX : MYB51-myc* #113 or 223 and *wrky33/DEX : WRKY33-flag* #313 or 424 as described in Barco et al. (2019b) with the following modification. Anti-FLAG M2 Affinity Gel (Sigma-Aldrich) and EZview™ Red Anti-c-Myc Affinity Gel (Sigma-Aldrich) were used to immunoprecipitate chromatin-bound MYB51-myc and WRKY33-flag, respectively.

Sequential ChIP was performed on WT/*DEX : MYB51-myc* #113 + *DEX : WRKY33-flag* #313 or 424 nuclear extracts as described in Mendoza-Parra et al. (2012) with modifications. Initial ChIP was performed using EZview™ Red Anti-c-Myc Affinity Gel. Chromatin was immunoprecipitated first with the c-Myc antibody and second with the FLAG antibody because of stronger chromatin binding with the c-Myc antibody. To avoid false positive IP readouts at the end of the assay (a common problem with sequential ChIP assays), the first antibody is covalently cross-linked to a solid substrate (Mendoza-Parra et al., 2012). Following washes, beads were incubated in TE containing 10 mM DTT for 30 min at 37˚C, and the released TF–DNA complexes were extracted into ChIP dilution buffer [1% Triton X-100, 1.2 mM EDTA, 16.7 mM Tris-Cl (pH 8), 167 mM NaCl, 1x Complete EDTA-free protease inhibitor cocktail (Roche)] and immunoprecipitated a second time using FLAG M2 antibody (Sigma-Aldrich) and Protein G magnetic beads (EMD Millipore, Burlington MA) pre-treated with 0.1% (w/v) non-fat milk in 1X PBS and 0.5 mg/ml sheared salmon sperm DNA.

PCR analysis was performed on nuclear extracts prior to antibody incubation (input) and after ChIP. PCR conditions were as follows: 95°C for 3 min; 40 cycles of 95°C for 15 s, 53°C for 15 s, and 72°C for 1 min; 72°C for 5 min. Primer sequences are listed in **Supplementary Table 2**. Densitometric determination of signal intensity in each ChIP and input sample was calculated using NIH ImageJ. Fold enrichment was determined by calculating the ratio of PCR product intensities in ChIP Dex/Mock to Input Dex/Mock. In cases where amplicons were absent, an arbitrary value of 10 was assigned.

### Bacterial Infection Assays and ETI Elicitations

*P. syringae* pv. *tomato* DC3000 (*Pst*) and *Pst avrRpm1* (*Psta*) were used for bacterial infection assays and ETI elicitations. A single colony of *Pst* was grown in 2 ml of LB medium containing 25 μg/ml rifampicin (Sigma-Aldrich). A single colony of *Psta* from a freshly streaked 3-day-old agar plate was grown in 50 ml of LB medium containing 25 μg/ml rifampicin and 50 μg/ml kanamycin (IBI Scientific, Peosta, IA). Both strains were grown to log phase, washed in sterile water twice or once, respectively, resuspended in sterile water to OD_600_ of 0.2, and incubated at room temperature with no agitation for 6 and ~2.5 h, respectively, prior to infection. Bacterial infection assays on 4- to 5-week-old adult leaves were performed as described in Barco et al. (2019b).

## Data Availability Statement

All datasets generated for this study are included in the article/**Supplementary Material.**

## Author Contributions

BB and NC performed pathogen assays, immunoblot assays, and ChIP experiments. BB performed all other experiments. BB and NC interpreted the results and wrote the paper.

## Conflict of Interest

The authors declare that the research was conducted in the absence of any commercial or financial relationships that could be construed as a potential conflict of interest.
