# Supplementary figures and images for: Hierarchical and Dynamic Regulation of Defense-Responsive Specialized Metabolism by WRKY and MYB Transcription Factors
Source: Front Plant Sci. 2020 Jan 31;10:1775. doi: 10.3389/fpls.2019.01775 (PMC7005594; doi:10.3389/fpls.2019.01775)

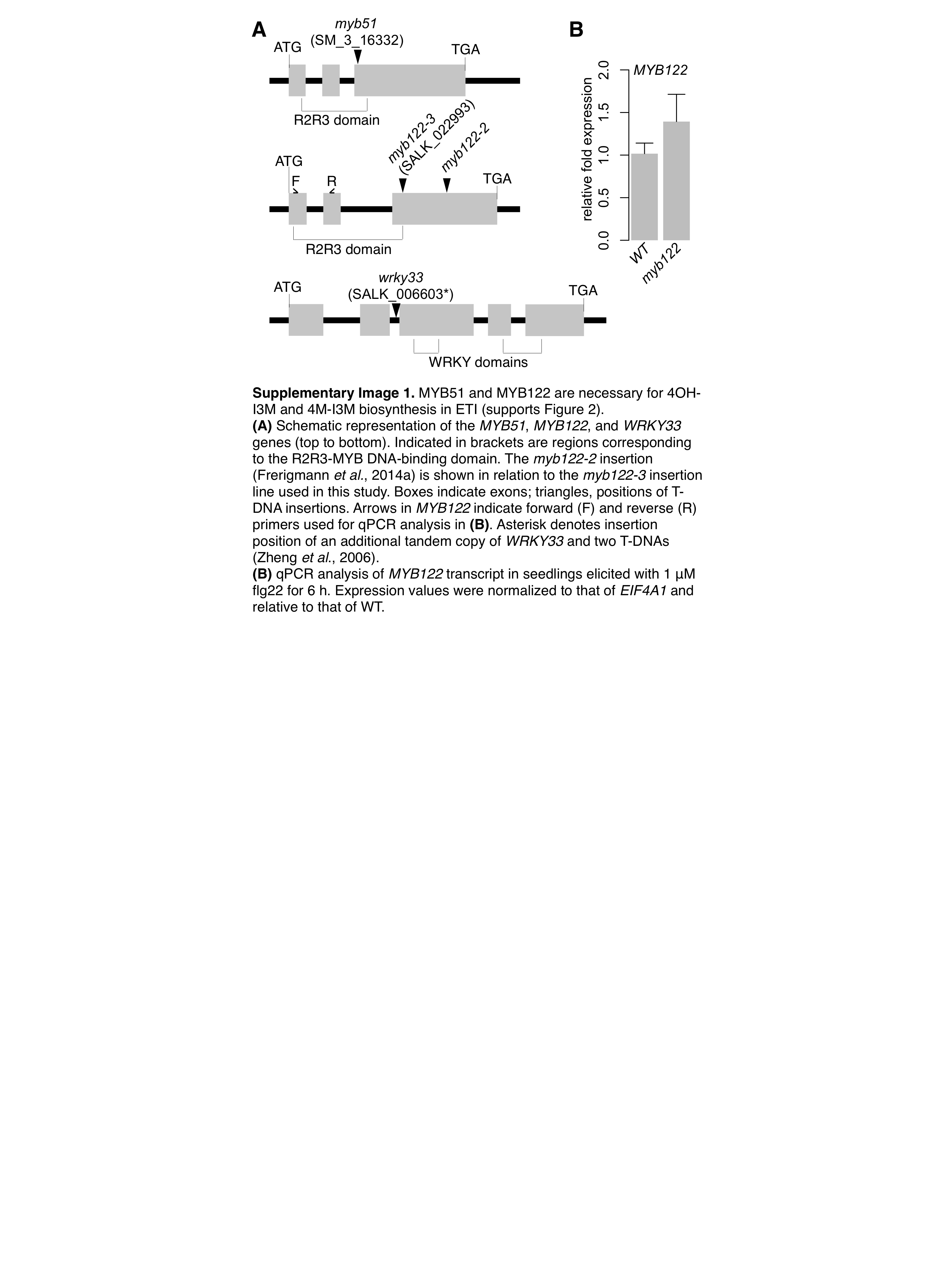

Supplement: Supplementary file 1 [file Image_1.jpg]

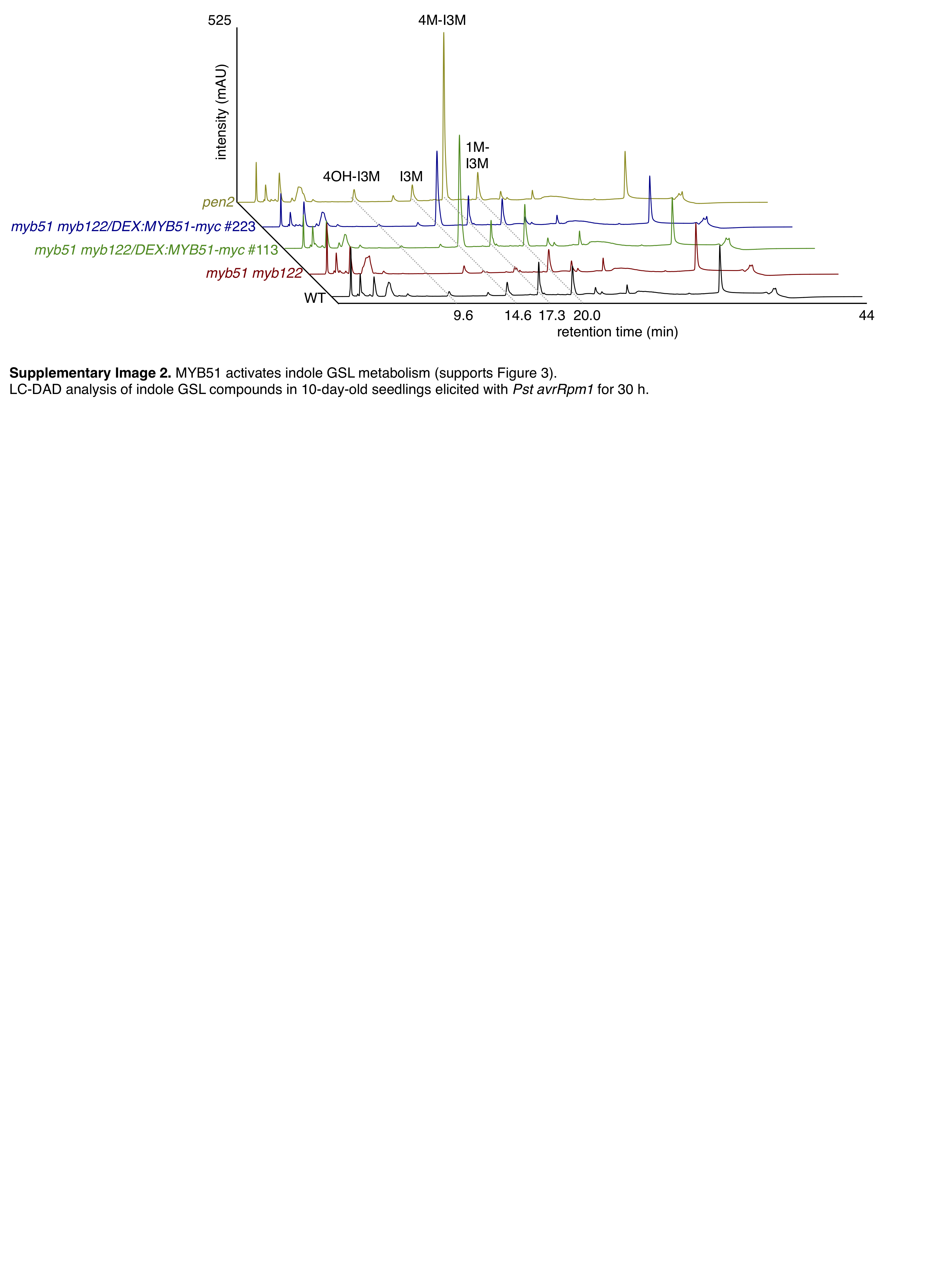

Supplement: Supplementary file 2 [file Image_2.jpeg]

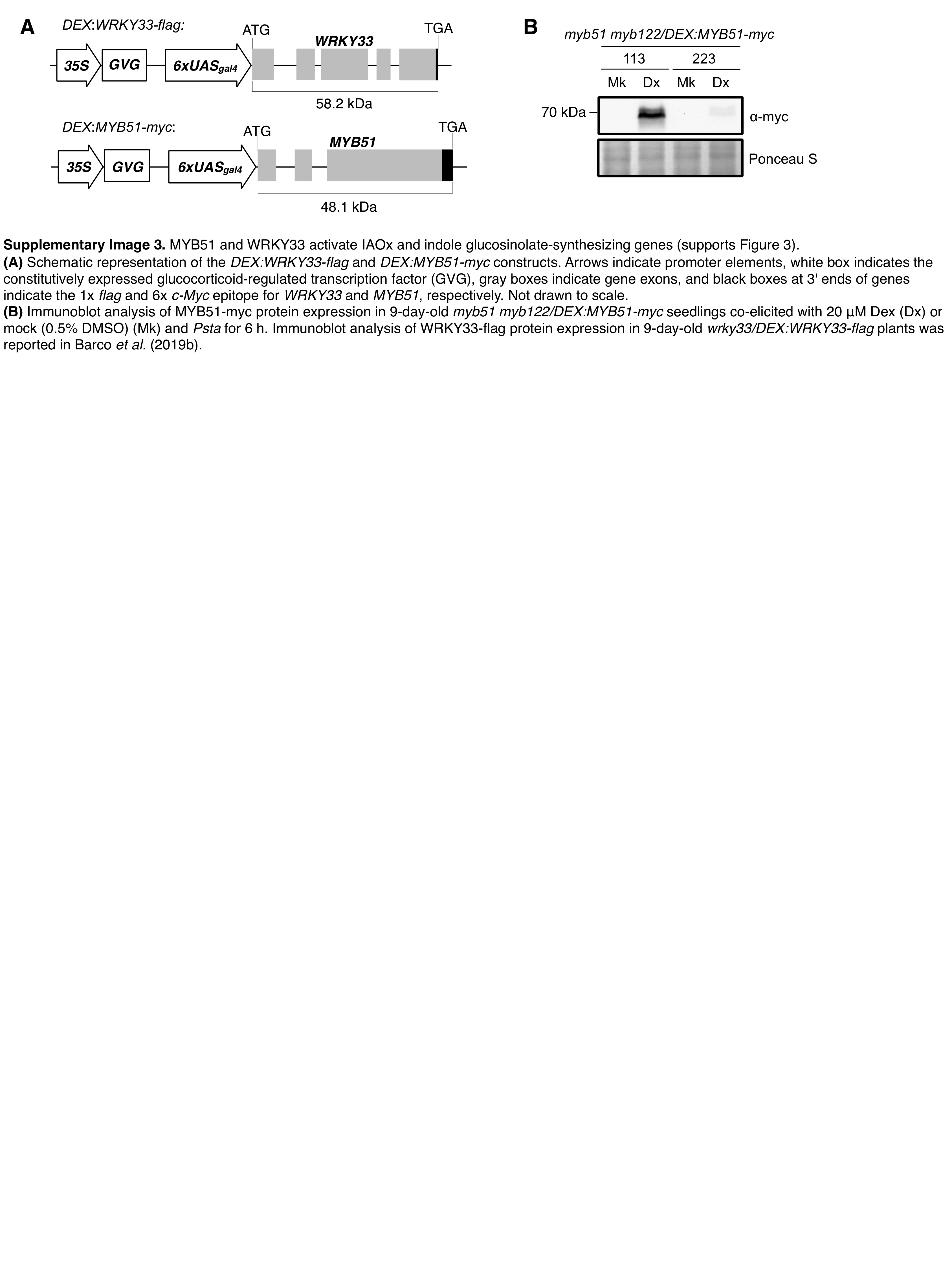

Supplement: Supplementary file 3 [file Image_3.jpg]

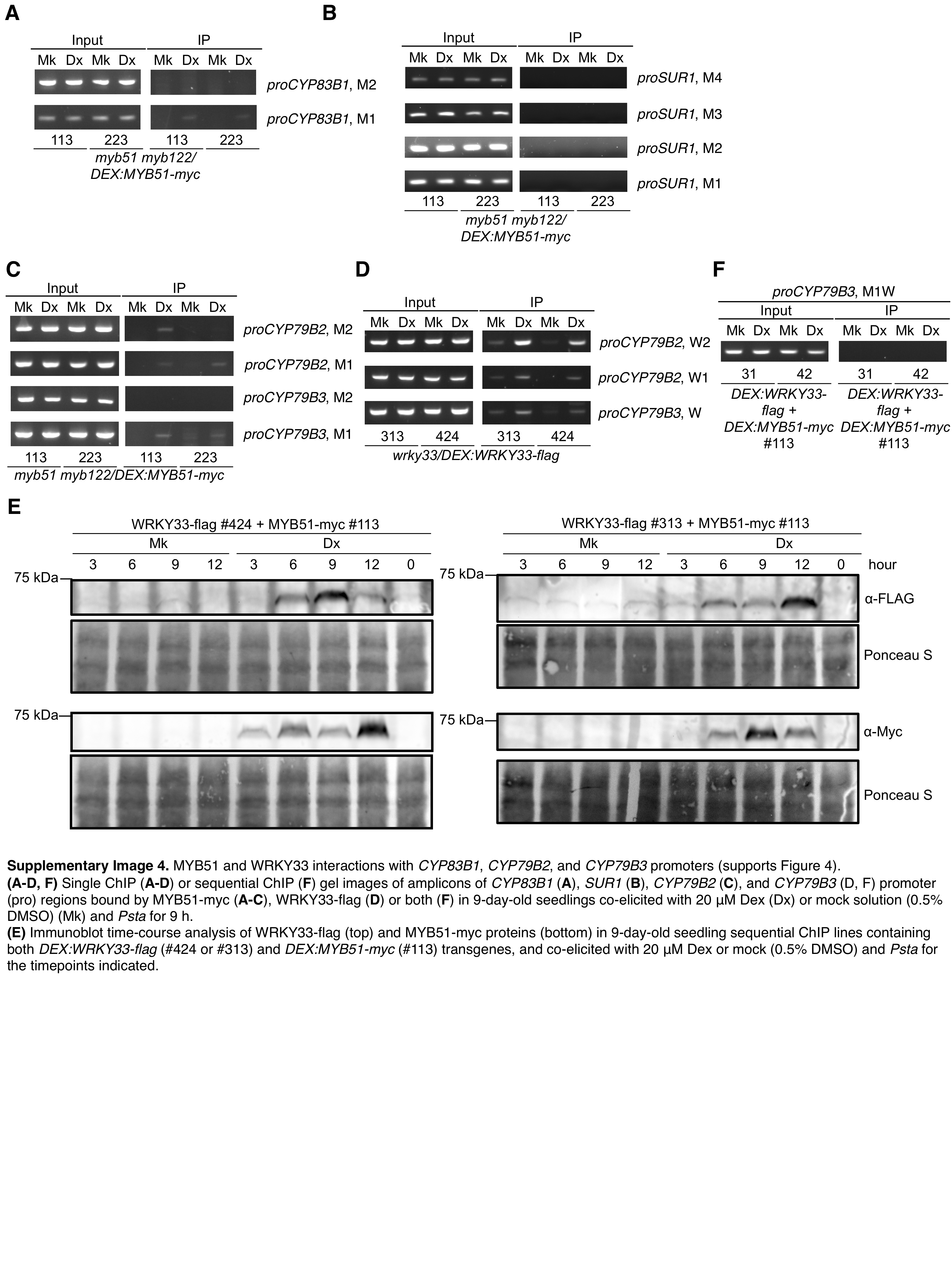

Supplement: Supplementary file 4 [file Image_4.jpg]

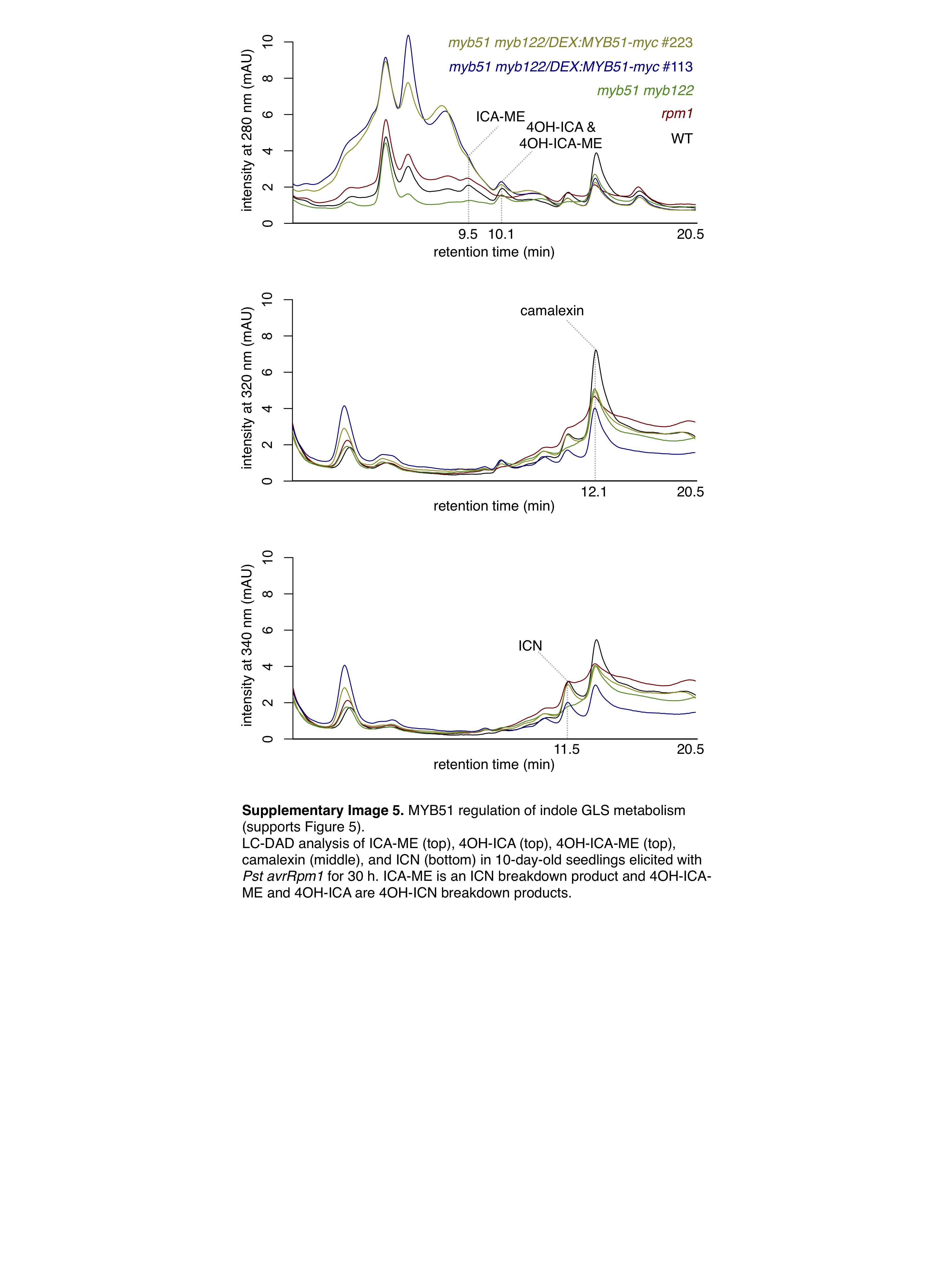

Supplement: Supplementary file 5 [file Image_5.jpeg]

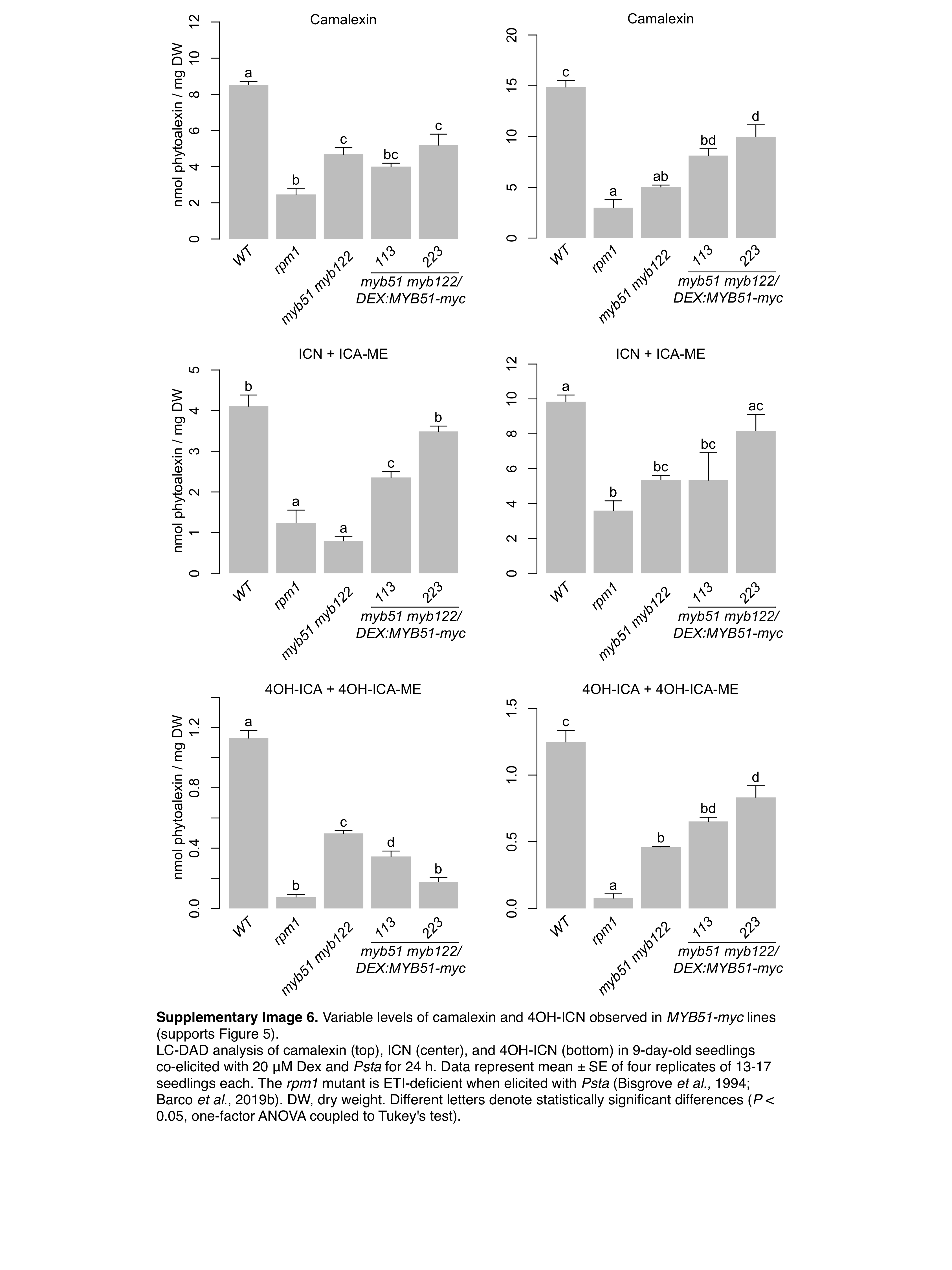

Supplement: Supplementary file 6 [file Image_6.jpg]

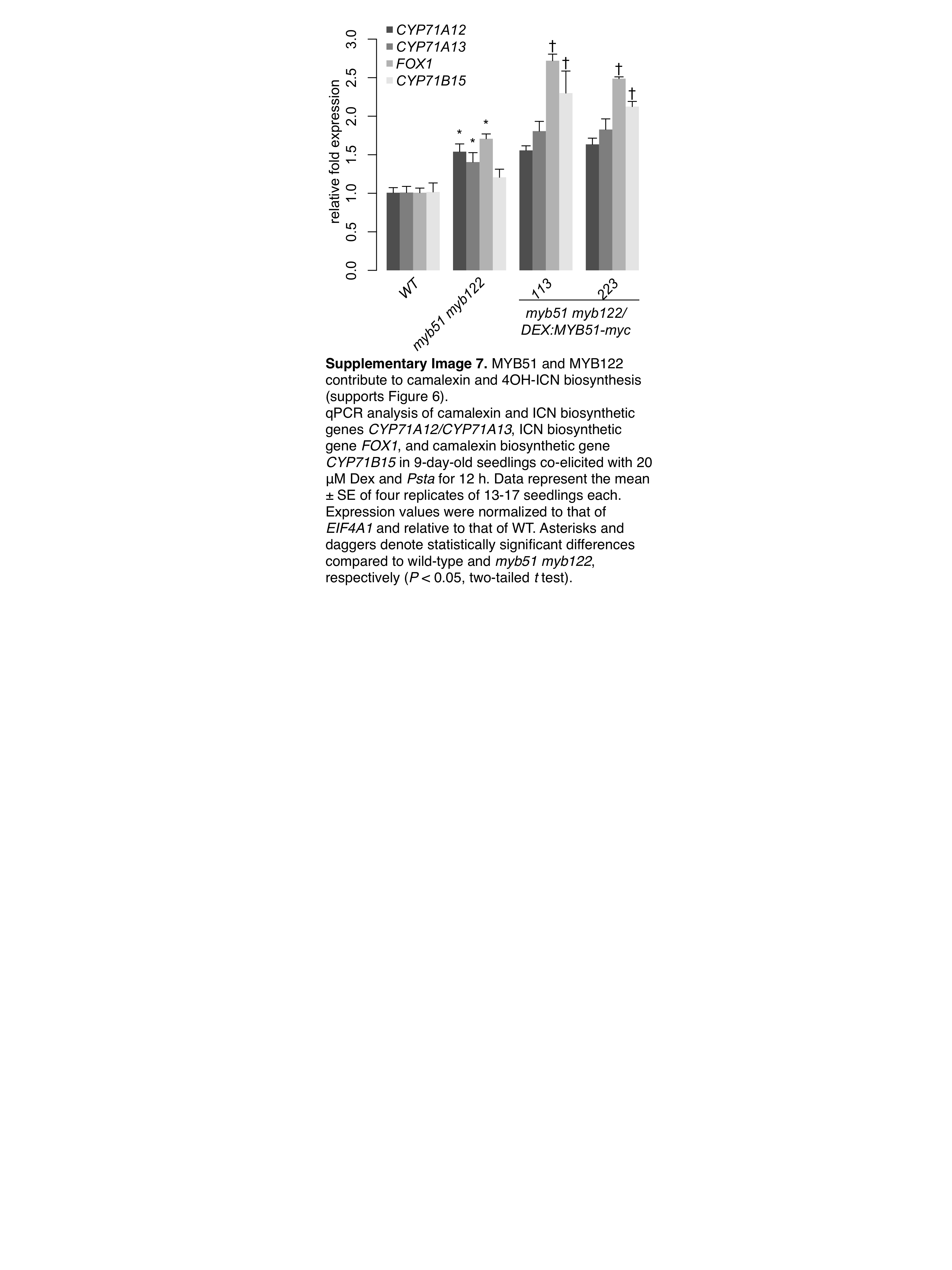

Supplement: Supplementary file 7 [file Image_7.jpg]

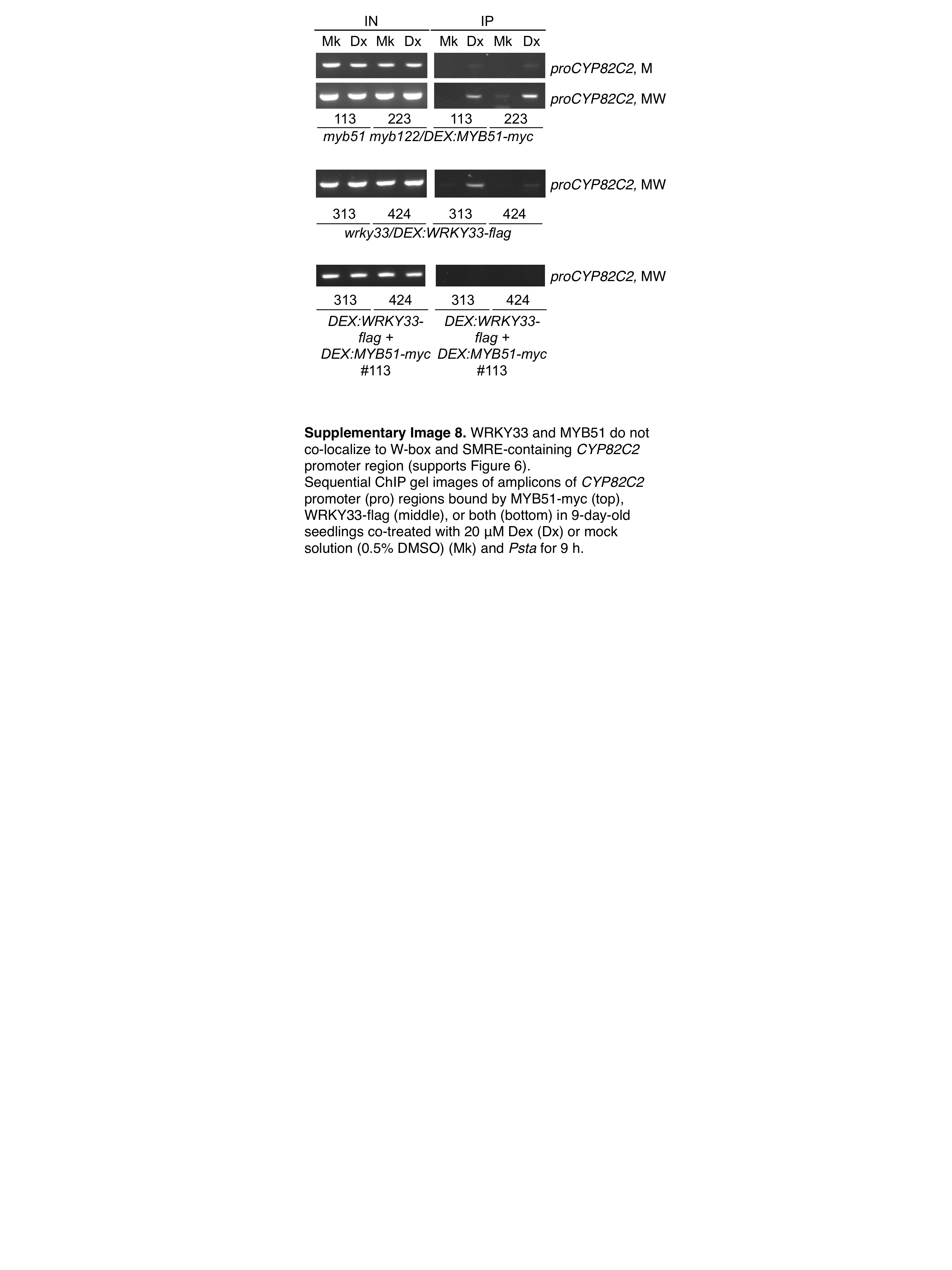

Supplement: Supplementary file 8 [file Image_8.jpg]

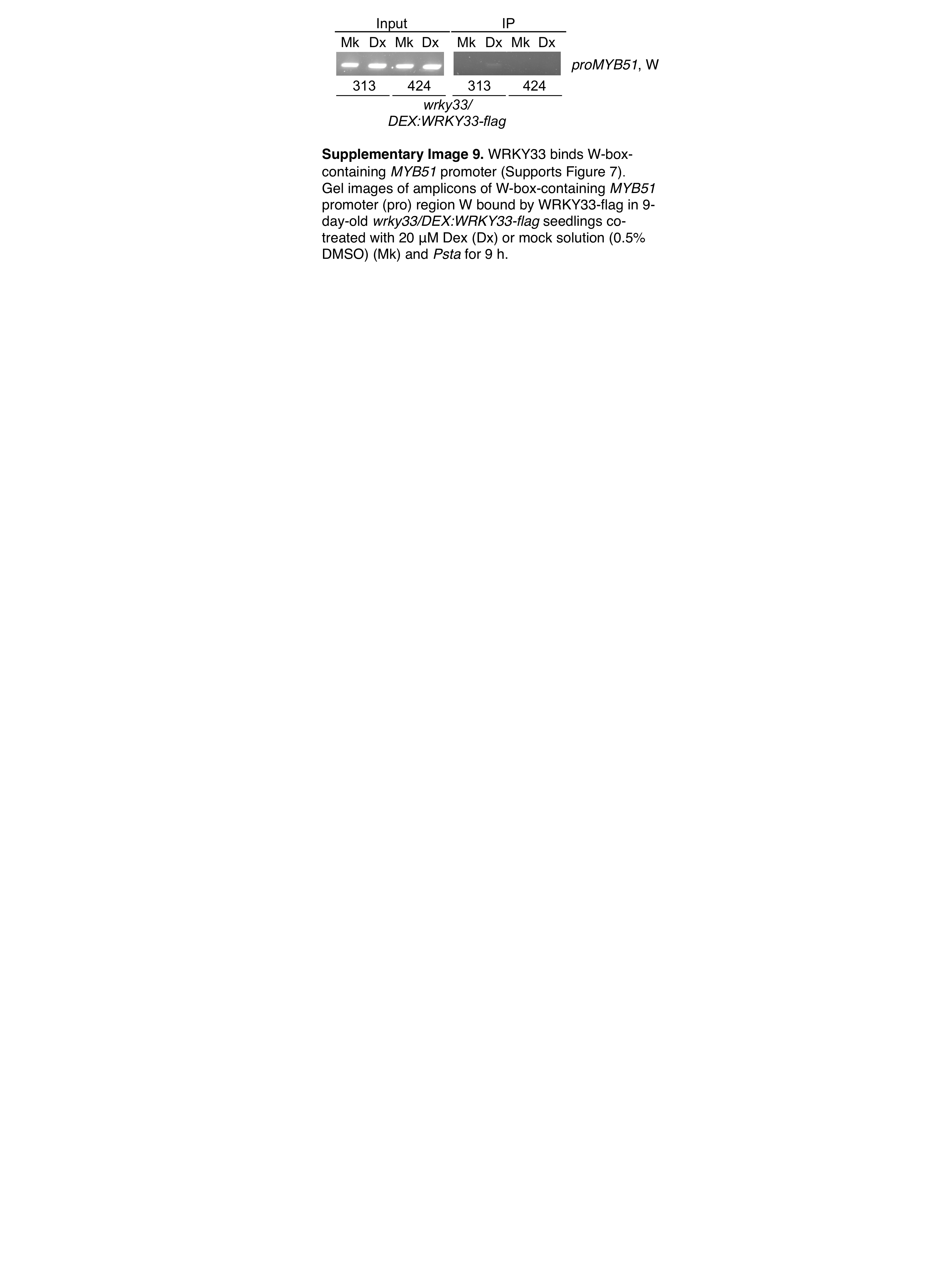

Supplement: Supplementary file 9 [file Image_9.jpg]
