# Supplementary material for: Hierarchical and Dynamic Regulation of Defense-Responsive Specialized Metabolism by WRKY and MYB Transcription Factors
Source: Front Plant Sci. 2020 Jan 31;10:1775. doi: 10.3389/fpls.2019.01775 (PMC7005594; doi:10.3389/fpls.2019.01775)
Supplement: Supplementary file 13 [file DataSheet_1.pdf]

W2, M2, W1, M1 promoter regions of *CYP79B2*

Agttagaatcgaattaatccaaaaggaattattgtggaattaactaaagcaatccatacaaatc
acaacaaaagctcgtcaagtatatataatcagaat**agtcaa(Wbox)**taatttagc**tttgaat(**
WRKY33)gccgaac**ttgact(Wbox)**aaaactcagacatgttatagtaccaaaatatttgtaat
ttagggaatatcattgctaatacaatataaaaaagatcatagctag**attcaaa(WRKY33)**atg
ttgaacatgacctacgacatt**gttgaat(WRKY33)**tcattgatctggtcttgctaaaacttt
aaaattgatgagttcaacatcttcaaatgcatgataacgggtccaacggaaa**ttgact(Wbox)**
tttttttcatgctcctgatatataataatatctaacgattacgggttccactaattgtcattac
tcattaacattcctatttaaaagttgtgatagttttagggttttacgtagtcgtgtcatatagc
gattaactacgtacttgtagatttatcaattacttctgttgtttacgagaacctaaaaaaaaga
agcagatgcctagtttatagagcacgtgtactgtcttgaaaactta**ggtaggt(SMRE8/ACI)**
tggtaaggttaccaaaagaccttaaaggaatataaagttactaattaacttaagtaa**agttggt**
(SMRE2)attgcttatatattgcaaagtattacaaaccaatcccctctgtatattgttttaaac
catagatttttttacaattaagtttatgatcaatcaattatttcaccatttctattaaattatg
taaaaagaaaaggatatatatatatatatataattaaataagaataaatcaaaataccgaaatt
ttttattatccattctttgtggacatcgcccctaatatataaaaaaaaaaaactttcgtataac
tgatttatattttttttgtaaaaacttaaaggaagcctaagaaatatcttgtgatattttttgaca
aaatgtggtatatatcttttttataatatcatttataaagaaaatattgattacatggtgaaaaa
cattttgctagcgatcaacaaaattaaataggcacatgttaactgatctcatacgaccttgaaa
ttttaatctttgtgtcgagagaccgatctttatgcaaattatgaaactacacatggtttatgca
cggaagatcacattgcatgtataccatattataaaccaaaaatgatcaagaagaaggcgaaaac
atttgggtaaattttaaatttcgatcatgcgatttttttagctcatcatcaacagacaagaaact
atcttttgtactgtaaatactaaatacaaaataaaatcttcatcatttttttgcatgtgtcaaat
aaattacgcgaacttttttttttttatcgactattaatagagaaacctgttttatttgccttgat
ttggaaaaatggagaaa**ttgact(Wbox)**taagacttagtctcggtcacatcggcaacaacgga
gcttaaacggcgtccgcaacatggaaactcaagccacgaatctgatata**ttgact(Wbox)**ata
gaagtagtaagtaact**ttgact(Wbox)**cgtccCacatcagtttcaatttccacgagggtattt
ggcaggtgaactctctacgtacccaaaacataatggctattttatttcataactgatatttagc
aattaattattcgtcctttttaaaccaatttctatagttgggaaaataatcaatttttacactt
tcaatgtatacgttacagattttttttttattagtcatgcacatattttcaatttttacactttc
aatgtaaacaatcgattcttaattgttaaaaataggtttacgtaaggaattaaagatttgttta
aaatatgttccggccggtctaataatttacttgacgttaatttcttaaacactttttagatagga
ggctttgtttatcccaaatgattttgtaccactgcgacaatactagctagacataaaatgttaa
taaattttttattaagtaatataatcgaagtattagatcaatgtagtagaca**gttaggt(SMRE7**
/ACIII)taactaaaacaagagtaaacacttttttttttcttttcaggataggtaaaacaaatt
tcacactattttgcgtatttccttaaatttgttgttcgttttctcagcaaagatgaatattttg
tttcatagtaattcacaagtataaactcgccagaactcctcaaacagtgaaatataatatagct
tttaactgtttttcggctggaccgggtttttaagtgcatatataacacgaggaattttggcagg
tcaccaacaaaactttaaaaatattaaaaattcccatcaagaatagaaattaataaacaatga
tatctctaataatatagatattttgaaacgttaggaataatcgtaataatgttcaacgttggtg
gtggtactcaagatggaccctccctcccacattttcctcactccttcgtaagtcctttccacgc
ataagggtattatagtcatttcacataaactaacgactactagacttgtatataaataggaagg
tgaagctctctctttatccatgcagagacaacagaaaccacaacaaaaactttgagtcctcttc
ttctctatacacaaac**ATG(TSS of CYP79B2)**

M2, **W**, ***M1W***, **and M1 promoter regions of *CYP79B3***

atataatttcaaatttacacattgtataacgatatgagatttacataaatattttcatatatt
tgttcattttaagagttcacaccacggataaattatcgatatataggaaagacttggctataac
tacaaaaagctgaagggacatttattattatccccaaaaaaagcaacatcatgttcgtattga
ttattgaatatgacatattgtagagacgaatcatttatgaagataaatcaaacg**acctacc(SM**
RE8/ACI) aaaataagatatactcaaaagagatataatgtaagttcaatcagttaaagctacta
ttatggctctgttagatatgagagtagtgctaagataaagagactaccaattcatattaccgag
aaaaaaaaaaactgtcttgttagtttagagtacatatataacacgaggattttggcaggtcac
caacaaaataattttaaaatatattatgaatgaattattatacatgattattatcttttat*gta*
*cattttttgaaagttagccctacaaa**attcaaa(WRKY33)**atatggtgacactcctcactaga*
*gacca**agttggt(SMRE2)**ccaactctagctataccttccacactgcttcc*tccccaccacaa
aaggggtactatcgtcatttcacacgt**acctacc(SMRE8/ACI)** tgtaattaatgtgacatta
taaataacaacgtgaaagaaggatccatgcagaaacaattagcatatattacaaaacacatact
aattgtttctccttctccttcttccttgcaa**ATG(TSS of CYP79B3)**

M and MW promoter region of *CYP82C2*

aggcctttgtctttgttactcacgacactgtcgttttgagtcttcgtttcgacttctccaaagg
gatcttgaagctgtctggattcagatcttgaaggtgatgtcggagcagtggaggtcatgctaat
atcaaca**accaaac(SMRE3)** ctgtaagcatatgagaagcttccaataaaacattcttaaaatt
tattttcaacaactgccaatgtattaaaaaaaagcaatctcatgaattgcctacacgctaatta
ggcgggcgaaatttgttttatgtgtatgtggtatggtatttgtttttcctcgccatcctttcct
ggtaaaaccaataatttttatatcacatataaagttacgtgctgaacgaaacaaccgattatt
attaagaatccatgttgaaatagagaaaatggattgtgtgaatagaaaaacaaaaacgaaaagt
cgacaaaaaaaacaaaaaacagaaaaacccactaa**agtcaa(Wbox**) gcgtttgtttcaacca
tgttatagtttagaactttataatcagaaattaattccacgaaaatgaataaatgtcagc**agtc**
aa(Wbox) accagaccattaaaagaaacaattaccattaaaagatata**ttgact(Wbox)** aaca
aaatcaatacaaacctttgcgtccatctctgttatggacacagttgacaattacaaaaaccctc
catcttcctgtctctctctatcatccatgacttttttttttctcaacaacaactaattattatc
caaagttcttagataatgagattaatacatgctatttcttgcccaactaggcacaatagaataa
agcttaggatcata**attcaaa**(**WRKY33)** aatgaaagagactcccttgatactctgccaatgtg
tgccctctcttggtatgaacacaaac**ttgacc(Wbox**) tcttcaaattgggggagcaacctctg
cagatcatccatgacttaaactgcacaataattaaaataggaaacttaagttcatcaagagaga
ccaaaaatatcacaaaactcttatttataccatttctattggcaatatttaatagtttttata
attaaaataataaaataaaagagaaaaaaagtaaaaaaattctcgcttgaatttttttgaaata
ctctatacaactcatagtgacattttttttttcaattggttcaatttttttttactaaaaaaa
aattttaattactttttttagaaattctttggggttctccttgatggaaaaagatataccaaaat
aggtaaagattcttgaacaatggtgtaccatctacgacttcaccgaagtgagctaaagcatccc
tttaacgttggtaatcagttacccatgacacacactggacatttaccttcatcactctggtcgt
cctgagacttgaacagtttcttgtttctgttatcatttacaacataaggcccacttcttgccca
cacacagtttattactcatcaatttctctaatatactattactctataacaacaaaaaagtaac
attgtaatttgtacacatagcgtttgatctagctagttctatatgtagaattgatgttagcaag
tgaatcaaaatttgcggagatgatgtttgaacatgggtttgttcattgtgttacccacacgaa
gccaatttgattgttcgtccagatccttgtaaaaagaacacatatggatcatttagtcgcaaca

ataaagaagatgtatcagagctacatatgtatgtttcacagtatttctcctctcgattaacaat
gaactggtgcaaggatcacccgattcgttcttgagggaagtatcttgggaagcagatcgattta
gaggcaaagagggctcaaaagaaatcgttgatgaaatcgctcaaggtctcgcttgaaggttcgc
agtttcttcactaatcgtgagcctttttaaagttgcagtaatcgttgtatgaaacaagcgtgca
cggtcatagtcatgctcgttttctcgaggcagcttgacaagagttaaattatggtgatggataa
ttagtgtatttttgaggctgtctcaactgaactttcatttaatgacttggctagcgatacagaa
ccgtctagccacaaaagagagattattaaagtggaatggcgaggcaaatggtggtagtgtgttt
tgtcaaacggaaatagaaacaagagatcacctatttttctcttgttcatactctcatgcttggt
cagctcttactatgaaatatatgctcggctctcgcttcacgacttgctgtgattctcttgttgc
cctatctacgacctttcctctccacaacttcatttgtttgtgtttagatatgtctttcaactc
actatacacaatctatggaaagaattaaatgccccataactcagagctcccacttctttttaaa
tcttgttctattaagttttcaaaatttctcggttttat**gttgaat(WRKY33**)ttattgtaaa
accgtt**tttgaat(WRKY33)**tttaaactcatacaaaaacaaaaaaatataattagtgtatttt
aaattatttgtgttttaatatattttttattttatcttttaaaacatgtttttttatttgagtta
ttattatgaattcagtattataaagtcatatcttcatttcaattttttt**tttgaat(WRKY33)**a
taatgttatataatatttctaaacacaagtagataacgtt**ggtcaa(Wbox)**t**atttggt(SMR
E1)**taagataaatggtggaaaaatattcagaaatgttcaaaaatgttcgaccattttttttatt
tcaaaatgtacgtcagtaactatcgatttttt**ttgacc(Wbox)**atatacaatttgcgaccccc
gcctttcgacgacttgctttt**ggtcaa(Wbox)**acagcagtaagataggcgtatgtctcatgct
tacatggtattgaaccgataatatgtgtgtgtatatatagagagacagactatacttttttaatc
attcaaa(WRKY33)actagaaatc**accaaac(SMRE3)**acacatctcttttgcacgctcaaac
cact**ATG(TSS of CYP82C2)**

M2 and M1 promoter regions of *CYP83B1*

aaacaaaggcttcaagaattctaccggaatcaATGGAGGTTCT**GGTAGGT(SMRE8/ACI)**TCT
ACGTTTAGGGATCGTAGCAGCGTCACGACGCATGATCAGGCCGTACCGGCGTCTCTGTCTAGCC
GGATTGGTTTGAGGAGATGTGGTAGATCTCCTCCGCCGGAGAGTTCGTCGTCCGTTGGAGAAAC
CAGCGAAAACGAGGAAGATGAAGACGACGCCGTTTCGTCATCACAAGGAAGATGGCTCAATTCG
TTTAGTTCATCTTTGGAAGATTCTCTTCCGATTAAgtacgtttctttattaattatcttctaat
tattctgttatctacacctgaatatattcgatcgatttattttattacaagaataaagtaaatc
attagattctcttatgttcttgggtttgattttattgatgaatctgtaagatgttgaaattgtt
tcttacttattagattctcagtttaaatgttattactacatgttgtgtttttgtttagtcatgt
ggtttaggttttggttttgttacagGAGAGGATTATCAAACCATTACATAGGGAAATCGAAAT
CGTTTGGGAATTTAATGGAAGCGAGTAACACAAACG**ATTTGGT(SMRE1)**GAAAGTGGAGAGTC
CATTGAACAAGAGAAGAAGATTGCTTATTGCTAATAAGCTAAGAAGAAGATCGTCCTTGTCTTC
TTTCAGCATCTACACTAAAATTAACCCTAATTCTATGCCGTTGCTTGCATTGCAAGAATCTGAC
AACGAGGATCATAAACTCAATGATGATGATGATGATGATGATAGTAGCAGTGATGATGAAACTA
GTAAGCTGAAAGAGAAGAGGATGAAGATGACGAACCATAGAGATTTTATGGTTCCTCAGACTAA
GAGCTGTTTTAGCTTAACTAGTTTTCAAGATGATGATGATCGATGAtcgatgatcattcttctt
cttttgtccaagagtagataaagtttcgttgacaaataagttggaggaagatttgacagaaacc
aatttacgtgtaattgtaataaattaatttgactgtttgtatattttgaattttctcaacgaag
tagactttttattttttttgtcacacttcttgtcaaccttaacatttgttagtaagcccatgag
attggttctataaattttaatgtccgcatgagaacaaagtcgttaatattgacggcttagtaat
ttcaatagcggctgcgaagtcaatagggacatctttaggctcgttctccgaaatactctttttt

tttttctctaccgagttgaatttatgtacgattcatcttagattgacgaagtagaatatttgt
ctcactgtg**acctact(SMRE6)**gttcaactgcaaaaatatgaacatatttttctacaaaaatt
gactacagagtaattagaaaacttaaaaactgagagtttagtttttggttacgtatactttttt
ttttctttcagtttacgttatagcataggagaaaatgatcgcttcaaatttcataggtattttt
aggaatttttctaaagtaccatttttatgctttgtttttaaatcgatcatatcagttcaaaaa
gaaaaaaatcaagaatgatgtctaaatgaacaaaagtttatgagatttataaaaaacaagttga
tgatatttattacttcataatatacttactgttctatagtatattttagaaaacaaaaccaa
gaggttatattcttatccaatatcttcacattttcaactcttcatcgatctcgctttcagtatt
attgtgtaacccaagttacattttttaattccaaaaaccagtagtcc**acctact(SMRE6)**aac
atcacgttttaaaaacaagggaaaattctgtcacctgtcaaaaaatccaaaacaa**accaacc(S
MRE4/ACII)**aataaaactttttgtcttgctatataaaccacatcatcattcaaagtagaaaag
tatccgaacacaaagacttaagtaagtcaaacagaaaaaa**ATG(TTS of CYP83B1)**

M4, M3, M2 and M1 promoter regions of *SUR1*

TTCCTTATCAAGAGTGTTTCTCTTATCTAGACTTGCAAAATCAATCAAATCTTGAGGAGAACAG
AGTGTATCATCACGTCCATCAAGAAGCCTTATAACTCGTATGAGTTCACTCACAACAATGGCCA
CCACACATCTTACAAAACACACATAAGAAAAAACAAAAGGAGTTAAAAAAGTGAAGAAAATATT
TCAATATCATATCTTATCTATATTACAATTAGAGTAGTTGTATTGTAAACTTTTCAATATTGTT
TTAAAGATTCTCATATGTACTTCATATATATGTCTCTTTATATTGCAGCATATAATAATCTTTT
CTCTACAAAATATTCATTCATATGTCCATTCCTTTTTCTCTTGCAACTTAACTGTGTTCTTTCT
GATCTCTTAAAGCTTTCATTATTATCCTTCAATCAGCTTTCAGTAAATCTGGGATTTTGAAATA
AAACTACAAGAACAATAATAAATTGTTAG**ACCAAAC(SMRE3)**TTGTTTCATAGAGAAAAAAAA
AAGAATCTGATACACTT**ACCTAAC(SMRE7/ACIII)**ATGTTGACCAAGATGCAAATGTAGATT
ATTTAATCGTCCTCCCAGCTCTCAAAAAAAAACCCATATCTTTAGCAATTTCTTTCAAAACATG
GAGAATATTTTTTCGAGCATCACTTAT**ACCAACC(SMRE4/ACII)**AGTGATTCAAGAAGCTGG
TGTAGTTCAAAAAGTATAGCCCTCACTCGTCGACGAACTTTAAATGAAAGTAATGAAAATCTGG
TATTAACTTGAAACTTTCACACTTCTCTTCCACTTTTATCCTTTCACACTAGAAAATTACAATG
TGCACAATTATAGCCGTTTCACACTTCTCTTCTACAATTTTTTTTTCACATCAAAAAATTATT
TCAGGTTTTTGACACATATTCCCAACAATTTATTTTTCTTAATAATTAATATAATTTTTAAAAT
ATATCAATGTACAAAACATAGGCTCCAAATGGATGACAAATAATTGAATATTTGGGTGCAAATG
GTTGCACAATTTAAAAGGTTGAAGAGGTTAAAAACATTATTCAATTAATCTTCAGCATTTTAGT
TGAAAAAGTTGAAAAAATTATTCAATTCATTCAACTTAAAAAGTTGAAAAATGAACAAATTATA
AAGGAAAAGTGTTCAACTTCTTGATCCACATTTTTGGAGATGATCATTCAACCCCAATTTTGTT
CA**ACCTAAT(SMRE5)**AATTTCAACTCATTCAATTTTGAAAAACCATTTGTACAAGTTGAAGAT
CTTTCAACTTATTCCTCTACAAAAAGGTTAAACAATTTTTCTTTATAACTTTTTTGGTTGAATG
GATGAAATTAGTTCTTAGTTATTTATCTCAAATAGTAAAAACATAAGATGAATGATCATTTAAC
TTTTTATAACCTCTTATTTTCAAACAACATTTGCACCCATAGTCTAAAAATAGAAATTTGTTGA
CCAAGAAGA**ATTTGGT(SMRE1)**ATCAAATGAATATTTTTTGTGGCTCAAACTTTTATTCTCTC
TTTAAGTAGCTGAAAAATTAACGTTGGAAAGTGGGGTCTACAGACCTTTACGCCCATTTATTTA
CGCTACAATGGTTTTCTAATCACATCCTTAATCTTACAATTTGTTTGATAATAAATCTACTAAT
TAAATTTGACGTCACAAATCACAATGCACTGACGCCCATTAGCATGTTAGTGACAATGTA**ACCA
AAC(SMRE3)**TGATTCATCACATTTCAAACTGAACTCATTCCGGTTGACTAATCGGTTGGGTTT
TAACTCGCCAACTATTCTTATCAGGCCAGGCCCAAACATTCCTTCACAAAGCCCTGCCAATTTG
ATTAATTTTTGTGGCACGTGATTTTAGTTTTGT**GTTAGGT(SMRE7/ACIII)**CTCACCAAGAT

TATTGATCACGTTCCATAATTCAAAACTTTGCATTATATACCAAAAATCCAATCCGTATTGAAA
CTTCATTTGTAAAGTTTTTTTTTTTCCCAAATAGTACTTTTTTTTTGTTTGGCCTTCTTATAGA
AAGAAGAACTCAAAGCACAGAGAGAAG**ATG(TTS of SUR1)**

W promoter region of *MYB51*

GattatttttTagtatttgtactaaagaactactgtaatttagttgtcatacttttagcatgaa
tcattgaatttagagttgggttgatgtaaaccaagctggttagctttcttcataaccgtatgta
ctttcatatcatattagagagaaaaaaaaacacaatg**ttgact(Wbox)**aattaatgggttctg
tctaactagaaaagtggaaattagttgtacgaaaaaagaaaatagcttatctctttctttgtac
cttagattttccttggtataatggatatcgatatgtccaagtccagtgtttgagtgaccaagt
tgttgctttaatttaatctagttcaaatacgactacttttgtgttgtacctctctaccttacct
tcctcgaattttcaattcgtgtgctagtttgccaattgtttctttataattttgtcgtctctat
gattgtttaagcatatttaaatatttgagagaaaaaaatactaagagttcagtcgtatttgaa
aagtcctcgcactccaagttttaaa**gttgaat(WRKY33)**tcattctgtcatggaaacgtacct
ttgtggtttttcatttcgatagacatttttaactcttctgataa**attcaaa(WRKY33)**atgt
tttttacattggttaaaatttgttgccaatttcaataaagctttttttttactcgttataaaa
ttaaaaacttgctgagaaattatagtttcatatatactttggaccgac**ggtcaa(Wbox)**tacc
aaaaaactaaaataaaatttt**ggtcaa(Wbox)**gaatcg**ggtcaa(Wbox)**tacaaaatatctc
caaagatcgaagagaaagttcaacaggattttaatttaattggtgttccggacctgccggcgcc
gtattataattcttcaataaatttatatttggattgcataatcaaaactatttgcatagcag
acatagatttcaaaataaattgatacgtaattagctatttacataggcaagacaattttacgtt
acatattttgcatgcttgatattcattcttttttttagcaacctttgctatattcattctatt
ttttcccctttttttgctactatcaatatcatatcagatgtggcggttcttatgcatgggatta
tgaaaaatgatattatataatagatgtcttcgtagtatttttttattgacaatcaaaataaaat
aaattagtttaagaaagatcaaaagtctagtgtcaaaatgtccactgacaaaagttagcagttt
ttcttttatgtaataatccgattataaaaaattgcgttaaggaatcataatgagccatgtgtca
gtcatatttaaacgacgacgttggcagcagccacacgtacagcctcaggcaagcataaatagag
atgggctctttcctataagtttcccactttgcaaagtgtaacacaaacagttctctttagagaa
aaaaagattctctctataaaccttctctttgtgtctctctttgtggtaagaaaacagagcaac
tagatctctcttctcctctgctcgttcaatgttataacgatctcgagtatcacaaaagaacaag
atgtctattcgaagaatctaccgatgtctgaattctttaatcaaaagatcttgtgctcaatctc
tatgatcatacgccccatttcacactttgtttcacctctcagtttttttttttgtgttttttg
tccctctttgtttcacttgagaacaaccccctttgaactcgatcaagaaagctaagtttgaaga
atcaaga**ATG(TTS of MYB51)**

Data sheet 1. Promoter regions amplified in ChIP-PCR experiments.
